# In silico SARS-CoV-2 vaccine development for Omicron strain using reverse vaccinology

**DOI:** 10.1007/s13258-022-01255-8

**Published:** 2022-06-04

**Authors:** Vladimir Li, Chul Lee, DongAhn Yoo, Seoae Cho, Heebal Kim

**Affiliations:** 1 Interdisciplinary Program in Bioinformatics, Seoul National University, Seoul, Republic of Korea; 2 Department of Agricultural Biotechnology, Research Institute for Agriculture and Life Sciences, Seoul National University, Seoul, Republic of Korea; 3 eGnome, Seoul, Republic of Korea

**Keywords:** SARS-CoV-2, Omicron, *In silico*, Reverse vaccinology, Vaccine design

## Abstract

**Background:**

The severe acute respiratory syndrome coronavirus 2 (SARS-CoV-2) pandemic began in 2019 but it remains as a serious threat today. To reduce and prevent spread of the virus, multiple vaccines have been developed. Despite the efforts in developing vaccines, Omicron strain of the virus has recently been designated as a variant of concern (VOC) by the World Health Organization (WHO).

**Objective:**

To develop a vaccine candidate against Omicron strain (B.1.1.529, BA.1) of the SARS-CoV-19.

**Methods:**

We applied reverse vaccinology methods for BA.1 and BA.2 as the vaccine target and a control, respectively. First, we predicted MHC I, MHC II and B cell epitopes based on their viral genome sequences. Second, after estimation of antigenicity, allergenicity and toxicity, a vaccine construct was assembled and tested for physicochemical properties and solubility. Third, AlphaFold2, RaptorX and RoseTTAfold servers were used to predict secondary structures and 3D structures of the vaccine construct. Fourth, molecular docking analysis was performed to test binding of our construct with angiotensin converting enzyme 2 (ACE2). Lastly, we compared mutation profiles on the epitopes between BA.1, BA.2, and wild type to estimate the efficacy of the vaccine.

**Results:**

We collected a total of 10 MHC I, 9 MHC II and 5 B cell epitopes for the final vaccine construct for Omicron strain. All epitopes were predicted to be antigenic, non-allergenic and non-toxic. The construct was estimated to have proper stability and solubility. The best modelled tertiary structures were selected for molecular docking analysis with ACE2 receptor.

**Conclusions:**

These results suggest the potential efficacy of our newly developed vaccine construct as a novel vaccine candidate against Omicron strain of the coronavirus.

**Supplementary information:**

The online version contains supplementary material available at 10.1007/s13258-022-01255-8.

## Introduction

Since its emergence in the late 2019 the COVID-19 resulted in 216 million infections with estimated 4.5 million of lethal cases by August 2021 (Weekly Epidemiological Update on COVID-19–31 August 2021 2021). Currently, five variants of concerns (VOC) of SARS-CoV 2 are circulating in mass according to the World Health Organization (WHO), including Alpha (B.1.1.7), Beta (B.1.351), Gamma (P.1), Delta (B.1.617.2) and the latest Omicron (B.1.1.529) variants (Tracking SARS-CoV-2 Variants 2021). Omicron variant B.1.1.529 of SARS-CoV-2 was designated as a VOC on November 26, 2021 (Update on Omicron 2022). Currently the information on its transmissibility, severity and vaccine effectiveness is yet to be confirmed. Currently two sublineages of B.1.1.529 exist including BA.1 and BA.2 (Statement on Omicron Sublineage BA.2 2022; Update on Omicron 2022).

Multiple vaccines were developed against the SARS-CoV-2, including Pfizer-BioNTech, Moderna, J&J Janssen, etc. (Self et al. 2021), and mainly used to prevent infections in various countries. However, a new strain, Omicron, was firstly identified on November 11, 2021 in Botswana and on November 14, 2021 in South Africa, and it can avoid neutralizing antibodies in vaccinated people by existing COVID vaccines (Cao et al. 2021). This situation is already predicted in a recent study among the US veterans shows that the SARS-CoV-2 vaccine effectiveness decreases (Cohn et al. 2021) and requires vaccine developments for the new strain. Standard process of vaccine development may take decades before a successful candidate is developed. To boost the development rate of vaccines, reverse vaccinology (RV) was developed. RV is a computational approach, which allows to discover the most potent vaccine candidates using bioinformatics methods, thus greatly amplifying the rate of vaccine development (Vaccine Research & Development 2022). Vaxign is a RV program, released in 2010, which was used to predict vaccine candidates for different pathogens (He et al. 2010). Vaxign2 and Vaxign-ML were developed to further promote the discovery of novel vaccines (Ong et al. 2021). As an example, reverse vaccinology was successfully used for the development of 4CMenB vaccine against meningococcal group B bacteria (Giuliani et al. 2022).

In this study, we utilized RV and other in silico methods to predict and assess a vaccine candidate for BA.1 and BA.2 subvariants of SARS-CoV-2 Omicron (B.1.1.529). For this purpose, we screened surface (S) glycoprotein and identified the most suitable epitopes. We then tested the selected epitopes for antigenicity and allergenicity to ensure vaccine safety. In the final step we performed molecular docking of the vaccine candidate with human ACE2 (Donoghue et al. 2000). As the result of this study, we developed a novel vaccine candidate against Omicron strain of the SARS-CoV-2.

## Materials and methods

### Sequence retrieval, preparation and screening of antigenic factor

Sequence of the spike(S) protein was retrieved from the reference sequence of SARS-CoV-2 in NCBI database (accession number: NC_045512) in FASTA format and mutations corresponding to SARS-CoV-2 Omicron subvariants BA.1 and BA.2 of B.1.1.529 were introduced to it (SARS-CoV-2 Variants-Stanford Coronavirus Antiviral & Resistance Database (CoVDB) 2022; Wu et al. 2020).

### MHC I and MHC II epitopes prediction

Vaxign 2 (http://www.violinet.org/vaxign2) was used to predict the adhesin probability and protective antigenicity of the selected candidate (Ong et al. 2021). The sequence was then submitted to Vaxitop server (http://www.violinet.org/vaxign2/vaxitop) to predict MHC I and MHC II epitopes (Ong et al. 2021). We also utilized the Immune Epitope Database (IEDB) to identify MHC I and MHC II epitopes to assess whether it would be able to produce results similar to Vaxitop (Bui et al. 2005, p. 2, 2005; Karosiene et al. 2012; Nielsen et al. 2003, 2007; Peters and Sette 2005; Sturniolo et al. 1999; Zhang et al. 2009).

### B cell epitopes prediction

ABCpred server (https://webs.iiitd.edu.in/raghava/abcpred/) was used to predict B cell epitopes (Saha and Raghava 2006). Default threshold and 16-mer length options were selected for the epitope prediction.

### Prediction of allergenicity, antigenicity and toxicity

First, selected epitopes were subjected to allergenicity test using AlgPred 2.0 server (https://webs.iiitd.edu.in/raghava/algpred2/). The threshold was set to 0.4 (Sharma et al. 2021). Second, antigenicity was predicted through VaxiJen server (http://www.ddg-pharmfac.net/vaxijen/VaxiJen/VaxiJen.html) with 0.4 threshold (Doytchinova and Flower 2007). These methods were performed in order to select epitopes, which would most likely trigger a strong immune response, while reducing to minimum the probability of allergenic (hypersensitivity) reactions. Third, the toxicity of MHC I, MHC II and B cell epitopes was predicted using ToxinPred server (https://webs.iiitd.edu.in/raghava/toxinpred/algo.php) (Gupta et al. 2013).

### Vaccine construction

**Fig. 1:**
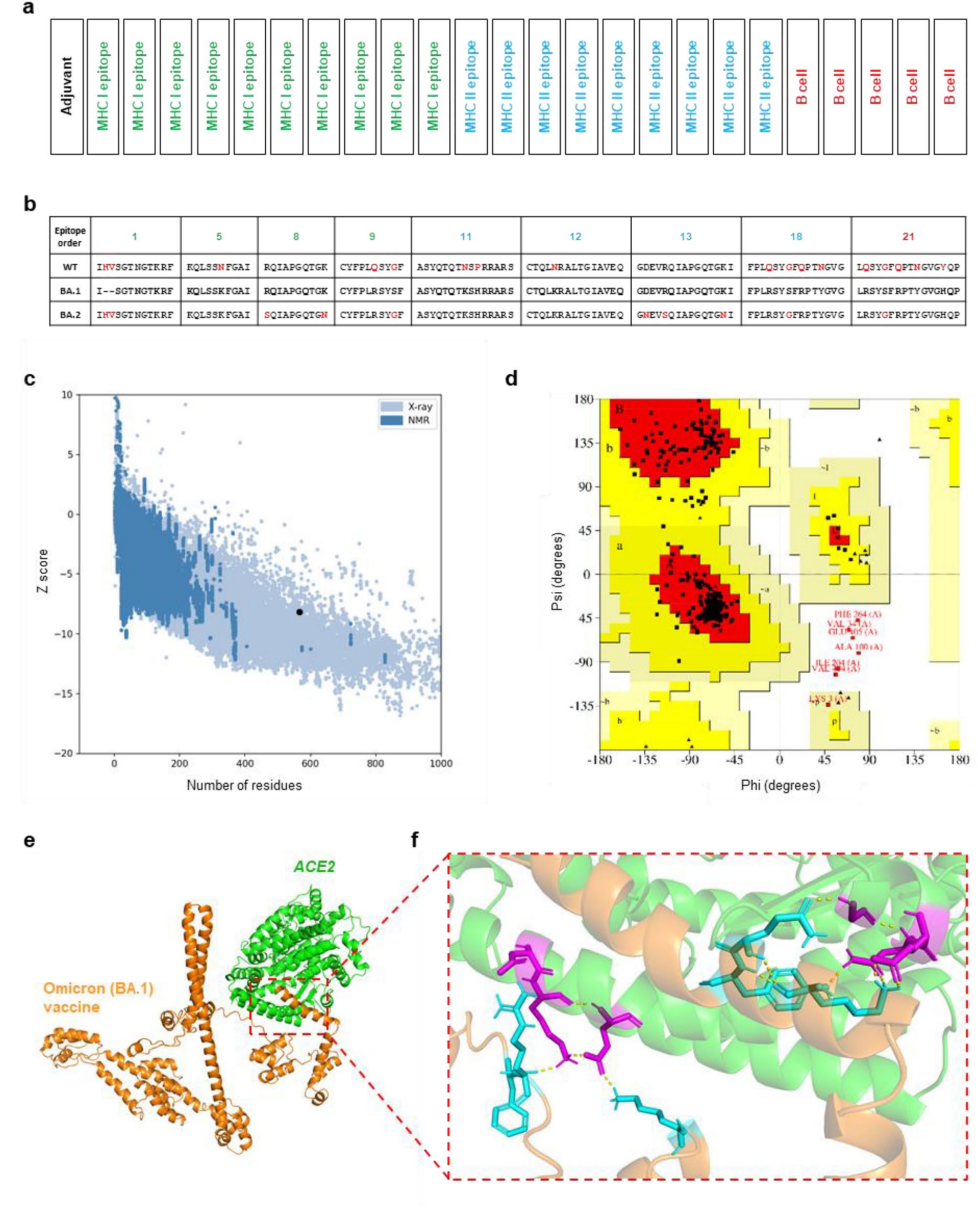
Omicron (BA.1) vaccine construct and its protein structure. The structure and analysis of the vaccine construct. **a** Schematic representation of the vaccine construct, **b** Mutation variations in epitope profiles, **c** Validation of the refined final model with ProSA-Web (Z-score = − 8.2), **d** Ramachandran plot of the vaccine model (93.7% of residues are located in the most favored regions and 1.2% of residues are located in disallowed regions), **e** Predicted 3D structure of the vaccine model in complex with ACE2 receptor, **f** Molecular docking visualizing hydrogen bonds between the vaccine model (cyan) and ACE2 (magenta) (color figure online)

The final vaccine construct was assembled by joining the adjuvant protein (50 S ribosomal protein L7/L12 (UniProt accession: P9WHE3)) with the selected epitopes through EAAAK linker (Arai et al. 2001; The UniProt Consortium 2019). The schematic representation of the vaccine construct along with epitope alignments between WT (NC_045512), BA.1 and BA.2 are presented on Fig. 1a; the linkers were omitted for simplicity.

### Physiochemical properties prediction and solubility

The physiochemical properties of the final construct were estimated using ProtParam web-server (https://web.expasy.org/protparam/) (Gasteiger et al. 2005). SOLpro (http://scratch.proteomics.ics.uci.edu/) was used to evaluate the solubility of the vaccine (Magnan et al. 2009).

### Protein secondary structure prediction

Protein secondary structure and solvent accessibility was predicted using RaptorX property (http://raptorx.uchicago.edu/StructurePropertyPred/predict/) server (Wang et al. 2016).

### Protein 3D structure prediction, refinement and structure validation

We utilized three web-servers (AlphaFold2, RaptorX and RoseTTAfold) to predict tertiary structures of our vaccine construct without using close homologs (Baek et al. 2021; Jumper et al. 2021; Mirdita et al. 2022; Peng and Xu 2011). AlphaFold is one of the newest protein folding tools created using artificial intelligence (AI) (Jumper et al. 2021; Mirdita et al. 2022). It demonstrated impeccable performance during 14th Critical Assessment of protein Structure Prediction (CASP) (Jumper et al. 2021). RaptorX server excels at predicting structures without close homologous structures in databases and was ranked the top in contact prediction in CASP12 and CASP13 (Peng and Xu 2011). RoseTTAfold is a tool, which utilizes neural network and simultaneously considers arrangement of amino acids, their interaction and possible tertiary structures they can form (Baek et al. 2021). PyMOL software was used to visualize the resulting protein (The PyMOL Molecular Graphics System, Version 2.0 2022). GalaxyRefine (http://galaxy.seoklab.org/) server was used to refine and relax the protein construct (Heo et al. 2013). The server consistently scores among the top algorithms in CASP. ProSA-Web server (https://prosa.services.came.sbg.ac.at/prosa.php) was used to validate the resulting protein structure (Wiederstein and Sippl 2007). This server compares the query model to structures in databases obtained through X-ray and NMR methods. PROCHECK server (https://saves.mbi.ucla.edu/) was used to generate a Ramachandran plot to identify allowed and disallowed psi and phi angles of amino acids within the model (Laskowski et al. 1993).

### Molecular docking

ACE2 (PDB: 6VW1) was retrieved from the Research Collaboration for Structural Bioinformatics (RCSB) Protein Data and used as a docking partner of the vaccine candidate (Shang et al. 2020). Docking between ACE2 receptor and the vaccine construct was performed in ClusPro 2.0 web-server (https://cluspro.bu.edu/) (Kozakov et al. 2017). ClusPro is based on PIPER algorithm, where receptor is presented as a stationary object and a ligand is rotated at different angles and positions with respect to it. The final step of docking is energy minimization using CHARMM. Third, PDBePISA (https://www.ebi.ac.uk/pdbe/pisa/) service was utilized to analyze protein-protein interface of the models (Krissinel and Henrick 2007).

## Results

### Epitope screening based on stability estimation for human

First, the protein sequence of Omicron S protein was uploaded to Vaxign 2 server for prediction of antigenic factor and epitope selection. The calculated Vaxign-ML score and adhesin probability were 96.1 and 0.533 correspondingly. The Vaxign-ML recommended threshold score of 90 indicates that the sequence has very high chances to trigger immune response in human organism (Ong et al. 2021). Neutralizing adhesins is important to prevent a pathogen entering host cells. The estimated value of 0.533 indicates adequate adhesin probability in the target protein sequence. Complete lists of epitopes predicted by Vaxitop and IEDB can be found is Supplementary data 8, 9 correspondingly.

Second, B cell epitopes were predicted utilizing ABCpred server. It uses artificial neural network in its algorithm and is suitable for identifying B cell linear epitopes for vaccine candidates (Saha and Raghava 2006). A total of 132 possible B cell epitopes were predicted, which were also sorted based on the scores produced by the server (Supplementary information 10).

We selected 10 MHC I, 9 MHC II and 5 B cell epitopes for vaccine construction based on the Vaxitop output by considering stability in human cells (Table 1). Selected epitopes were also screened for being antigenic, non-toxic and non-allergenic using the above-mentioned methods (Table 2). The antigenicity was calculated by the VaxiJen server, where epitopes’ antigenicity was predicted by alignment free approach based on auto cross covariance (ACC) (Doytchinova and Flower 2007). The values above 0.4 were deemed as antigenic (Supplementary information 1). Epitopes’ toxicity was predicted by ToxinPred server using support vector machine (SVM) (Supplementary information 1). Non-allergenic epitopes were predicted via AlgPred 2 server by using machine learning approach (Random Forest) and Basic Local Alignment Search Tools (BLAST) and motif-emerging and with classes-identification (MERCI) algorithms (Sharma et al. 2021). Selected threshold of 0.4 ensured that epitopes with values below it was assumed as non-allergenic (Supplementary information 1). The majority of selected MHC I and MHC II epitopes from Vaxitop were also identified in IEDB output, and, therefore, were selected for the final vaccine construct (Supplementary information 2). The vaccine constructs of BA.2 subvariant for both Vaxign 2 and IEDB were created by aligning the corresponding epitopes of our BA.1 constructs with the S protein sequence.

**Table 1:** Physiochemical properties of the vaccine construct (BA.1)

#	Properties	Results
1	Number of amino acids	567
2	Molecular weight	60 kDa
3	Acidic amino acids	67
4	Basic amino acids	77
5	Chemical formula	C_2676_H_4296_N_726_O_814_S_11_
6	Estimated half-life in *E. coli*	> 10 h
7	Estimated half-life in mammalian cells	30 h
8	Estimated half-life in yeast	> 20 h
9	Instability index (II)	35.24
10	Aliphatic index	81.55
11	GRAVY	− 0.163
12	Solubility upon overexpression	0.95
13	Alpha-helix (H)	50%
14	Beta-sheet (E)	6%
15	Coil (C)	43%

**Table 2:** Selected MHC class I and MHC class II epitopes and their properties

	Epitope	Antigenicity	Start	End	Allergenicity	Toxicity
MHC I	ISGTNGTKRF	0.5526	68	79	Non-Allergen	Non-toxic
	FPNITNLCPF	1.3964	329	338	Non-Allergen	Non-toxic
	KFLPFQQFGR	0.441	558	567	Non-Allergen	Non-toxic
	KIYSKHTPI	0.7455	202	210	Non-Allergen	Non-toxic
	KQLSSKFGAI	0.568	964	973	Non-Allergen	Non-toxic
	LIDLQELGKY	0.7076	1197	1206	Non-Allergen	Non-toxic
	LPIGINITRF	1.3027	229	238	Non-Allergen	Non-toxic
	RQIAPGQTGK	1.7893	408	417	Non-Allergen	Non-toxic
	CYFPLRSYSF	1.5062	488	497	Non-Allergen	Non-toxic
	DISGINASV	0.4155	1168	1176	Non-Allergen	Non-toxic
MHC II	ASYQTQTKSHRRARS	0.7544	672	686	Non-Allergen	Non-toxic
	CTQLKRALTGIAVEQ	0.7763	760	774	Non-Allergen	Non-toxic
	GDEVRQIAPGQTGKI	0.9741	404	418	Non-Allergen	Non-toxic
	SAIGKIQDSLSST	0.5434	929	941	Non-Allergen	Non-toxic
	SECVLGQSKRVDFCGKGYHL	0.9179	1030	1049	Non-Allergen	Non-toxic
	CALDPLSETKCTLKSFTVEK	0.5593	291	310	Non-Allergen	Non-toxic
	TISVTTEILPVSMT	1.2621	719	732	Non-Allergen	Non-toxic
	FPLRSYSFRPTYGVG	0.7464	490	504	Non-Allergen	Non-toxic
	DCLGDIAARDLI	0.4525	839	850	Non-Allergen	Non-toxic
B cell	GVSVITPGTNTSNQVA	0.4651	594	609	Non-Allergen	Non-toxic
	LRSYSFRPTYGVGHQP	0.4532	492	507	Non-Allergen	Non-toxic
	HRSYLTPGDSSSGWTA	0.6017	245	260	Non-Allergen	Non-toxic
	TRFQTLLALHRSYLTP	0.5115	236	251	Non-Allergen	Non-toxic
	YEQYIKWPWYIWLGFI	0.951	1206	1221	Non-Allergen	Non-toxic

### Physiochemical stability prediction

Physiochemical properties of the vaccine candidate were predicted by two independent programs (Table 2). ProtParam estimated molecular weight as 60 kDa and the instability index (II) as 35.24, indicating a stable protein structure (Gasteiger et al. 2005). The grand average of hydropathicity (GRAVY) index was calculated at − 0.163. GRAVY index measures average hydrophobicity and hydrophilicity of a protein based on the amino acids content. Negative values indicate that a protein is hydrophilic, while positive values indicate its hydrophobicity (Chang and Yang 2013). SOLpro server predicted the construct to be soluble with a probability of 0.95. The complete list of physicochemical properties of all constructed models can be found in Supplementary information 4.

### Protein structure prediction, refinement and validation

The models with the highest proportion of residues in most favored regions and the best Z-score were selected for further refinement. GalaxyRefine server was used to refine the selected tertiary structure (Supplementary information 5). We used ProSA-Web server and Ramachandran plot to validate the refined protein structure. Model produced by RoseTTAfold outperformed models created by the other servers. The calculated Z-score was − 8.2. (Fig. 1c). The Ramachandran plot demonstrated that 93.7% of amino acid residues were located in the most favored regions, (Fig. 1d). The complete list with all values can be found in Supplementary information 6.

### Molecular docking

ClusPro 2.0 web-server was utilized to simulate receptor-vaccine interaction (Fig. 1e, f). To maximize docking performance, we supplied information about contact residues of ACE2 (PDB: 6VW1) to the server (Kozakov et al. 2017; Shang et al. 2020). PDBePISA database was used to analyze interacting interfaces of the vaccine construct and the receptor. The results indicated that the solvation free energy (Δ^i^G) of the interacting partners was equal to − 11.5 kcal/mol, indicating that our vaccine construct makes stronger interaction with the ACE2 (Supplementary information 7). In addition, we also performed molecular docking analysis with the top scoring models produced by AlphaFold2 and RaptorX (Supplementary information 6).

## Discussion

Previously vaccine candidates for COVID-19 were developed using computational approaches, however, they were based on the original SARS-CoV-2 protein sequences and thus do not guarantee to develop protective immunity against Omicron strain (Singh et al. 2020; Tahir ul Qamar et al. 2020). In this study we proposed a new vaccine candidate against Omicron strain SARS-CoV-2 S protein using reverse vaccinology approach. We prepared S protein of Omicron strain by introducing the corresponding mutations and deletions to the original SARS-CoV-19 sequence (NC_045512) (SARS-CoV-2 Variants - Stanford Coronavirus Antiviral & Resistance Database (CoVDB) 2022; Wu et al. 2020). To our knowledge, this is the first vaccine design study developed specifically to reduce the spread of Omicron strain of the coronavirus infection.

In this work we attempted to design vaccine construct based on BA.1 and BA.2 subtypes of Omicron (B.1.1.529) strain of SARS-CoV-2. When designing vaccines, it is important to consider viral subtypes so the vaccines can have wider specificity. Since subtypes BA.1 and BA.2 have various mutations we concluded that comparing vaccine effectiveness would be complicated due to different epitopes profile of the constructs (SARS-CoV-2 Variants - Stanford Coronavirus Antiviral & Resistance Database (CoVDB) 2022). Thus, we used the epitopes of BA.1 predicted by Vaxign2 and IEDB as templates for BA.2 subtype via alignment of the S proteins. The safety of a vaccine is the first thing to consider, thus epitope used in the construct should be non-toxic and non-allergenic. When these criteria are satisfied, the epitopes should demonstrate adequate immunogenic properties to trigger immune response in the host. We predicted MHC I and MHC II epitopes using Vaxign2, ensuring their antigenicity, non-toxicity and non-allergenicity to construct a vaccine. Vaxign 2 and Vaxign-ML demonstrated the best results in predicting antigens over software such as Vaxijen3 and Antigenic, and thus were selected for this study (Ong et al. 2021). Moreover, an adjuvant was added to the final vaccine construct. It is a component of a vaccine which can increase immune response to an antigen (Guideline on Adjuvants in Vaccines for Human Use 2005). *M. tuberculosis* 50 S ribosomal protein L7/L12 is a toll-like receptor (TLR) 4 agonist, which promote dendritic cell maturation and induce T cell-mediated-cytotoxic response (Lee et al. 2014). SARS-CoV-2 was shown to have a potency to activate TLR4, thus modulating this receptor can increase chances to trigger immune response against the construct. Based on these results we assume that the vaccine construct has high chances of inducing proper immune response.

In order to investigate the potential efficacy of Omicron vaccine against other SARS-CoV-19 strains, we compared the mutation profiles on epitopes based on viral genomic sequences of BA.1, BA.2 and WT (Fig. 1b). Out of total 24 epitopes, 15 epitopes (62.5%) showed perfectly identical peptide sequences across all three strains. The others (37.5%) had 16 and 10 mutations on 7 and 6 epitopes in WT and BA.2, respectively, when compared to the BA.1 subtype as a reference. It suggests BA.1 vaccine can have the efficacy for other strains via the larger proportions of epitopes without mutations. Focusing on the other epitopes with mutations, BA.2 can have a stronger potentiality to trigger protective immunity than WT, because of less number of mutations harboring small number of epitopes. We believe that subtype specific mutations present in the selected epitopes will be sufficient in triggering immune response against BA.1 and epitopes shared between all three instances can also potentially contribute to the protective immunity against various strains of SARS-CoV-19 if they could not accumulate sufficient substitutions on the epitopes to avoid the Omicron vaccine.

Tertiary structures of our vaccine constructs were evaluated by Ramachandran plot and Z-score estimated by ProSA-Web server and the top performing models were selected for further analysis. Overall, models demonstrated high proportion of residues in allowed regions and Z-scores comparable with the range of scores (X-ray, NMR) normally found within native proteins of the similar size (Laskowski et al. 1993; Wiederstein and Sippl 2007). In this study, RaptorX and RoseTTAfold produced better models than AlphaFold2. Both Raptor X and RoseTTAfold demonstrated well-estimated protein folding with adequate proportion of residues in most favorable and disallowed regions with reliable Z-scores. On the other hands, AlphaFold2 generated worse models, although, had the highest number of residues in favorable regions and the lowest proportion of residues in disallowed regions showed very poor Z-score in a range 3.7–5.4 for all models. This indicates that tertiary structures were not folded properly and thus were removed from assessment. Visual inspection also confirmed that the structures had relatively high proportion of unfolded regions.

The molecular docking indicated positive interaction of the vaccine construct with the ACE2, mimicking virus interaction with the receptor. These results indicate the possibility for eliciting immune response against the viral epitopes presented by the constructs. However, despite the high theoretical chances of our models to be a vaccine candidate, our results are a theoretical estimation and require *in vitro*/*in vivo* confirmation of the results to make sure the vaccine is safe and effective before making solid conclusions.

## Electronic supplementary material

Below is the link to the electronic supplementary material.

Supplementary Material 1

Supplementary Material 2
