# Supplementary material for: In silico SARS-CoV-2 vaccine development for Omicron strain using reverse vaccinology
Source: Genes Genomics. 2022 Jun 4;44(8):937–44. doi: 10.1007/s13258-022-01255-8 (PMC9166176; doi:10.1007/s13258-022-01255-8)
Supplement: Supplementary file 1 — Supplementary Material 1 [file 13258_2022_1255_MOESM1_ESM.docx]

**Supplementary information 1.** Selected MHC I and MHC II epitopes (Vaxign 2) used in this study.

| **BA. 1** | | | | | | | | | | | | | | |  |
| --- | --- | --- | --- | --- | --- | --- | --- | --- | --- | --- | --- | --- | --- | --- | --- |
| **MHC I** | | | | | | | | | | | | | | |  |
| **Vaxign2** | | | | | |  | **Algpred** | |  | **Vaxijen** | |  | **ToxinPred** | | |
| **Epitope** | **MHC allele** | **Start** | **End** | **Length** | **P-value** |  | **Score** | **Prediction** |  | **Score** | **Prediction** |  | **SVM score** | **Prediction** | |
| ISGTNGTKRF | HLA-A*01:01 | 68 | 79 | 10 | 0.001 |  | 0.35 | Non-Allergen |  | 0.5526 | Antigen |  | -0.95 | Non-Toxin | |
|  | HLA-A*29:02 |  |  |  | 0.08 |  |  |  |  |  |  |  |  |  |  |
|  | HLA-A*30:02 |  |  |  | 0.048 |  |  |  |  |  |  |  |  |  |  |
|  | HLA-B*15:01 |  |  |  | 0.091 |  |  |  |  |  |  |  |  |  |  |
| FPNITNLCPF | HLA-B*35:01 | 329 | 338 | 10 | 0 |  | 0.38 | Non-Allergen |  | 1.3964 | Antigen |  | -0.89 | Non-Toxin | |
|  | HLA-B*07:02 |  |  |  | 0.004 |  |  |  |  |  |  |  |  |  |  |
|  | HLA-B*15:01 |  |  |  | 0.071 |  |  |  |  |  |  |  |  |  |  |
| KFLPFQQFGR | HLA-A*31:01 | 558 | 567 | 10 | 0 |  | 0.4 | Non-Allergen |  | 0.441 | Antigen |  | -0.84 | Non-Toxin | |
|  | HLA-A*68:01 |  |  |  | 0.019 |  |  |  |  |  |  |  |  |  |  |
|  | HLA-A*33:01 |  |  |  | 0.014 |  |  |  |  |  |  |  |  |  |  |
| KIYSKHTPI | HLA-B*08:01 | 202 | 210 | 9 | 0.021 |  | 0.35 | Non-Allergen |  | 0.7455 | Antigen |  | -1.09 | Non-Toxin | |
|  | HLA-B*15:01 |  |  |  | 0.026 |  |  |  |  |  |  |  |  |  |  |
|  | HLA-B*58:01 |  |  |  | 0.034 |  |  |  |  |  |  |  |  |  |  |
|  | HLA-A*32:01 |  |  |  | 0 |  |  |  |  |  |  |  |  |  |  |
|  | HLA-C*07:02 |  |  |  | 0.019 |  |  |  |  |  |  |  |  |  |  |
|  | HLA-C*08:02 |  |  |  | 0.041 |  |  |  |  |  |  |  |  |  |  |
|  | HLA-C*14:02 |  |  |  | 0.005 |  |  |  |  |  |  |  |  |  |  |
|  | HLA-C*15:02 |  |  |  | 0.045 |  |  |  |  |  |  |  |  |  |  |
|  | HLA-A*02:11 |  |  |  | 0.006 |  |  |  |  |  |  |  |  |  |  |
|  | HLA-A*69:01 |  |  |  | 0.008 |  |  |  |  |  |  |  |  |  |  |
|  | HLA-A2 |  |  |  | 0.001 |  |  |  |  |  |  |  |  |  |  |
|  | HLA-B*57:01 |  |  |  | 0.068 |  |  |  |  |  |  |  |  |  |  |
|  | HLA-B7 |  |  |  | 0.022 |  |  |  |  |  |  |  |  |  |  |
|  | HLA-C*03:03 |  |  |  | 0.008 |  |  |  |  |  |  |  |  |  |  |
|  | HLA-C*05:01 |  |  |  | 0.042 |  |  |  |  |  |  |  |  |  |  |
|  | HLA-C*06:02 |  |  |  | 0.036 |  |  |  |  |  |  |  |  |  |  |
|  | HLA-A*02:12 |  |  |  | 0.006 |  |  |  |  |  |  |  |  |  |  |
|  | HLA-A*02:16 |  |  |  | 0.013 |  |  |  |  |  |  |  |  |  |  |
|  | HLA-A*02:19 |  |  |  | 0.001 |  |  |  |  |  |  |  |  |  |  |
|  | HLA-A*02:50 |  |  |  | 0.019 |  |  |  |  |  |  |  |  |  |  |
|  | HLA-A*32:07 |  |  |  | 0.06 |  |  |  |  |  |  |  |  |  |  |
|  | HLA-B*27:20 |  |  |  | 0.011 |  |  |  |  |  |  |  |  |  |  |
| KQLSSKFGAI | HLA-A*02:01 | 964 | 973 | 10 | 0.009 |  | 0.34 | Non-Allergen |  | 0.568 | Antigen |  | -0.54 | Non-Toxin | |
|  | HLA-A*02:02 |  |  |  | 0.012 |  |  |  |  |  |  |  |  |  |  |
|  | HLA-A*02:03 |  |  |  | 0.008 |  |  |  |  |  |  |  |  |  |  |
|  | HLA-A*02:06 |  |  |  | 0 |  |  |  |  |  |  |  |  |  |  |
|  | HLA-B*44:02 |  |  |  | 0.019 |  |  |  |  |  |  |  |  |  |  |
|  | HLA-A*24:02 |  |  |  | 0.05 |  |  |  |  |  |  |  |  |  |  |
|  | HLA-A2 |  |  |  | 0.041 |  |  |  |  |  |  |  |  |  |  |
|  | HLA-B*15:01 |  |  |  | 0.027 |  |  |  |  |  |  |  |  |  |  |
| LIDLQELGKY | HLA-A*01:01 | 1197 | 1206 | 10 | 0 |  | 0.3 | Non-Allergen |  | 0.7076 | Antigen |  | -0.91 | Non-Toxin | |
|  | HLA-A*29:02 |  |  |  | 0.035 |  |  |  |  |  |  |  |  |  |  |
|  | HLA-A*30:02 |  |  |  | 0.002 |  |  |  |  |  |  |  |  |  |  |
| LPIGINITRF | HLA-B*53:01 | 229 | 238 | 10 | 0 |  | 0.38 | Non-Allergen |  | 1.3027 | Antigen |  | -1.04 | Non-Toxin | |
|  | HLA-B*35:01 |  |  |  | 0.006 |  |  |  |  |  |  |  |  |  |  |
|  | HLA-B*07:02 |  |  |  | 0.01 |  |  |  |  |  |  |  |  |  |  |
| RQIAPGQTGK | HLA-A*03:01 | 328 | 337 | 10 | 0 |  | 0.35 | Non-Allergen |  | 1.7893 | Antigen |  | -0.79 | Non-Toxin | |
|  | HLA-A*11:01 |  |  |  | 0 |  |  |  |  |  |  |  |  |  |  |
|  | HLA-A*31:01 |  |  |  | 0.004 |  |  |  |  |  |  |  |  |  |  |
|  | HLA-A*68:01 |  |  |  | 0.026 |  |  |  |  |  |  |  |  |  |  |
|  | HLA-B*27:05 |  |  |  | 0.008 |  |  |  |  |  |  |  |  |  |  |
| CYFPLRSYSF | HLA-A*23:01 | 488 | 497 | 10 | 0.001 |  | 0.32 | Non-Allergen |  | 1.5062 | Antigen |  | -0.58 | Non-Toxin | |
|  | HLA-A*24:02 |  |  |  | 0.008 |  |  |  |  |  |  |  |  |  |  |
|  | HLA-A*29:02 |  |  |  | 0.075 |  |  |  |  |  |  |  |  |  |  |
| DISGINASV | HLA-A*02:01 | 1168 | 1176 | 9 | 0.027 |  | 0.32 | Non-Allergen |  | 0.4155 | Antigen |  | -0.94 | Non-Toxin | |
|  | HLA-A*68:02 |  |  |  | 0.001 |  |  |  |  |  |  |  |  |  |  |
|  | HLA-B*08:01 |  |  |  | 0.046 |  |  |  |  |  |  |  |  |  |  |
|  | HLA-B*51:01 |  |  |  | 0.034 |  |  |  |  |  |  |  |  |  |  |
|  | HLA-A*26:01 |  |  |  | 0.007 |  |  |  |  |  |  |  |  |  |  |
|  | HLA-A*69:01 |  |  |  | 0.004 |  |  |  |  |  |  |  |  |  |  |
|  | HLA-A2 |  |  |  | 0.004 |  |  |  |  |  |  |  |  |  |  |
|  | HLA-B*57:01 |  |  |  | 0.049 |  |  |  |  |  |  |  |  |  |  |
|  | HLA-A*32:07 |  |  |  | 0.035 |  |  |  |  |  |  |  |  |  |  |
| **MHC II** | | | | | | | | | | | | | | |  |
| **Vaxign2** | | | | | |  | **Algpred** | |  | **Vaxijen** | |  | **ToxinPred** | | |
| **Epitope** | **MHC allele** | **Start** | **End** | **Length** | **P-value** |  | **Score** | **Prediction** |  | **Score** | **Prediction** |  | **SVM score** | **Prediction** | |
| ASYQTQTKSHRRARS | HLA-DPA1*01:03/DPB1*02:01 | 672 | 686 | 15 | 0 |  | 0.3 | Non-Allergen |  | 0.7544 | Antigen |  | -1.24 | Non-Toxin | |
|  | HLA-DRB1*10:01 |  |  |  | 0.011 |  |  |  |  |  |  |  |  |  |  |
|  | HLA-DPA1*01:03/DPB1*04:01 |  |  |  | 0.001 |  |  |  |  |  |  |  |  |  |  |
| CTQLKRALTGIAVEQ | HLA-DRB1*01:01 | 760 | 774 | 15 | 0.016 |  | 0.35 | Non-Allergen |  | 0.7763 | Antigen |  | -1.38 | Non-Toxin | |
|  | HLA-DRB5*01:01 |  |  |  | 0.02 |  |  |  |  |  |  |  |  |  |  |
|  | HLA-DRB1*08:02 |  |  |  | 0.028 |  |  |  |  |  |  |  |  |  |  |
|  | HLA-DRB1*13:02 |  |  |  | 0.008 |  |  |  |  |  |  |  |  |  |  |
| GDEVRQIAPGQTGKI | HLA-DRB1*04:01 | 404 | 418 | 15 | 0.006 |  | 0.37 | Non-Allergen |  | 0.9741 | Antigen |  | -0.76 | Non-Toxin | |
|  | HLA-DRB1*09:01 |  |  |  | 0.01 |  |  |  |  |  |  |  |  |  |  |
|  | HLA-DPA1*02:01/DPB1*05:01 |  |  |  | 0.018 |  |  |  |  |  |  |  |  |  |  |
| SAIGKIQDSLSST | HLA-DRB4*01:01 | 929 | 941 | 13 | 0 |  | 0.35 | Non-Allergen |  | 0.5434 | Antigen |  | -1.28 | Non-Toxin | |
|  | HLA-DRB5*01:01 |  |  |  | 0.004 |  |  |  |  |  |  |  |  |  |  |
|  | HLA-DRB1*07:01 |  |  |  | 0.022 |  |  |  |  |  |  |  |  |  |  |
|  | HLA-DRB1*11:01 |  |  |  | 0.002 |  |  |  |  |  |  |  |  |  |  |
|  | HLA-DRB1*15:01 |  |  |  | 0.007 |  |  |  |  |  |  |  |  |  |  |
| SECVLGQSKRVDFCGKGYHL | HLA-DRB1*01:01 | 1030 | 1049 | 20 | 0.009 |  | 0.34 | Non-Allergen |  | 0.9179 | Antigen |  | -0.09 | Non-Toxin | |
|  | HLA-DRB1*04:01 |  |  |  | 0.016 |  |  |  |  |  |  |  |  |  |  |
|  | HLA-DR15 |  |  |  | 0.005 |  |  |  |  |  |  |  |  |  |  |
|  | HLA-DRB1*15:01 |  |  |  | 0 |  |  |  |  |  |  |  |  |  |  |
| CALDPLSETKCTLKSFTVEK | HLA-DR15 | 291 | 310 | 20 | 0.008 |  | 0.39 | Non-Allergen |  | 0.5593 | Antigen |  | -1.22 | Non-Toxin | |
| TISVTTEILPVSMT | HLA-DRB1*04:01 | 719 | 732 | 14 | 0 |  | 0.26 | Non-Allergen |  | 1.2621 | Antigen |  | -1.34 | Non-Toxin | |
| FPLRSYSFRPTYGVG | HLA-DQA1*01:01/DQB1*05:01 | 490 | 504 | 15 | 0.027 |  | 0.32 | Non-Allergen |  | 0.7464 | Antigen |  | -1.13 | Non-Toxin | |
| DCLGDIAARDLI | HLA-DRB1*01:01 | 839 | 850 | 12 | 0.001 |  | 0.36 | Non-Allergen |  | 0.4525 | Antigen |  | -0.71 | Non-Toxin | |

**Supplementary information 2.** Selected MHC I and MHC II epitopes (IEDB) used in this study.

| **BA. 1** | | | | | | | | | | | | | | | |
| --- | --- | --- | --- | --- | --- | --- | --- | --- | --- | --- | --- | --- | --- | --- | --- |
| **MHC I** | | | | | | | | | | | | | | | |
| **IEDB** | | | | | | |  | **Algpred** | |  | **Vaxijen** | |  | **ToxinPred** | |
| **Epitope** | **MHC allele** | **Start** | **End** | **Length** | **Score** | **Percentile rank** |  | **Score** | **Prediction** |  | **Score** | **Prediction** |  | **SVM score** | **Prediction** |
| ISGTNGTKRF | HLA-B*57:01 | 68 | 79 | 10 | 0.26263 | 1.1 |  | 0.35 | Non-Allergen |  | 0.5526 | Antigen |  | -0.95 | Non-toxic |
|  | HLA-B*58:01 |  |  |  | 0.131467 | 1.1 |  |  |  |  |  |  |  |  |  |
|  | HLA-B*15:01 |  |  |  | 0.009416 | 5.4 |  |  |  |  |  |  |  |  |  |
|  | HLA-A*30:02 |  |  |  | 0.006681 | 8.1 |  |  |  |  |  |  |  |  |  |
|  | HLA-A*01:01 |  |  |  | 0.005365 | 5.4 |  |  |  |  |  |  |  |  |  |
|  | HLA-A*24:02 |  |  |  | 0.00222 | 5.9 |  |  |  |  |  |  |  |  |  |
|  | HLA-A*32:01 |  |  |  | 0.001478 | 8.8 |  |  |  |  |  |  |  |  |  |
|  | HLA-A*23:01 |  |  |  | 0.001344 | 7.4 |  |  |  |  |  |  |  |  |  |
|  | HLA-B*53:01 |  |  |  | 0.000836 | 11 |  |  |  |  |  |  |  |  |  |
|  | HLA-A*26:01 |  |  |  | 0.000715 | 13 |  |  |  |  |  |  |  |  |  |
|  | HLA-B*51:01 |  |  |  | 0.000627 | 22 |  |  |  |  |  |  |  |  |  |
|  | HLA-A*31:01 |  |  |  | 0.00053 | 20 |  |  |  |  |  |  |  |  |  |
|  | HLA-B*35:01 |  |  |  | 0.000475 | 14 |  |  |  |  |  |  |  |  |  |
|  | HLA-A*68:01 |  |  |  | 0.000421 | 20 |  |  |  |  |  |  |  |  |  |
|  | HLA-B*44:02 |  |  |  | 0.000313 | 14 |  |  |  |  |  |  |  |  |  |
|  | HLA-A*30:01 |  |  |  | 0.00029 | 43 |  |  |  |  |  |  |  |  |  |
|  | HLA-B*08:01 |  |  |  | 0.000271 | 33 |  |  |  |  |  |  |  |  |  |
|  | HLA-B*44:03 |  |  |  | 0.00013 | 19 |  |  |  |  |  |  |  |  |  |
|  | HLA-A*33:01 |  |  |  | 0.000127 | 33 |  |  |  |  |  |  |  |  |  |
|  | HLA-B*07:02 |  |  |  | 0.000124 | 29 |  |  |  |  |  |  |  |  |  |
|  | HLA-A*02:06 |  |  |  | 0.0001 | 44 |  |  |  |  |  |  |  |  |  |
|  | HLA-A*11:01 |  |  |  | 0.000092 | 22 |  |  |  |  |  |  |  |  |  |
|  | HLA-A*03:01 |  |  |  | 0.000089 | 30 |  |  |  |  |  |  |  |  |  |
|  | HLA-A*68:02 |  |  |  | 0.000037 | 49 |  |  |  |  |  |  |  |  |  |
|  | HLA-A*02:03 |  |  |  | 0.000035 | 50 |  |  |  |  |  |  |  |  |  |
|  | HLA-B*40:01 |  |  |  | 0.000031 | 30 |  |  |  |  |  |  |  |  |  |
|  | HLA-A*02:01 |  |  |  | 0.000027 | 51 |  |  |  |  |  |  |  |  |  |
| FPNITNLCPF | HLA-B*35:01 | 329 | 338 | 10 | 0.581019 | 0.18 |  | 0.38 | Non-Allergen |  | 1.396 | Antigen |  | -0.89 | Non-toxic |
|  | HLA-B*53:01 |  |  |  | 0.454515 | 0.16 |  |  |  |  |  |  |  |  |  |
|  | HLA-B*07:02 |  |  |  | 0.17679 | 0.75 |  |  |  |  |  |  |  |  |  |
|  | HLA-B*51:01 |  |  |  | 0.094779 | 1.4 |  |  |  |  |  |  |  |  |  |
|  | HLA-B*08:01 |  |  |  | 0.012794 | 5.2 |  |  |  |  |  |  |  |  |  |
|  | HLA-A*26:01 |  |  |  | 0.002181 | 7.5 |  |  |  |  |  |  |  |  |  |
|  | HLA-B*15:01 |  |  |  | 0.0021 | 11 |  |  |  |  |  |  |  |  |  |
|  | HLA-B*57:01 |  |  |  | 0.00145 | 16 |  |  |  |  |  |  |  |  |  |
|  | HLA-A*01:01 |  |  |  | 0.00087 | 16 |  |  |  |  |  |  |  |  |  |
|  | HLA-A*02:06 |  |  |  | 0.000832 | 21 |  |  |  |  |  |  |  |  |  |
|  | HLA-A*24:02 |  |  |  | 0.000825 | 8.7 |  |  |  |  |  |  |  |  |  |
|  | HLA-B*58:01 |  |  |  | 0.000675 | 16 |  |  |  |  |  |  |  |  |  |
|  | HLA-A*23:01 |  |  |  | 0.000612 | 11 |  |  |  |  |  |  |  |  |  |
|  | HLA-A*30:02 |  |  |  | 0.000406 | 35 |  |  |  |  |  |  |  |  |  |
|  | HLA-A*68:02 |  |  |  | 0.000201 | 29 |  |  |  |  |  |  |  |  |  |
|  | HLA-A*68:01 |  |  |  | 0.000158 | 27 |  |  |  |  |  |  |  |  |  |
|  | HLA-A*02:03 |  |  |  | 0.000125 | 34 |  |  |  |  |  |  |  |  |  |
|  | HLA-B*44:02 |  |  |  | 0.00012 | 21 |  |  |  |  |  |  |  |  |  |
|  | HLA-A*32:01 |  |  |  | 0.000115 | 25 |  |  |  |  |  |  |  |  |  |
|  | HLA-B*44:03 |  |  |  | 0.000111 | 21 |  |  |  |  |  |  |  |  |  |
|  | HLA-A*33:01 |  |  |  | 0.000093 | 37 |  |  |  |  |  |  |  |  |  |
|  | HLA-A*30:01 |  |  |  | 0.000041 | 72 |  |  |  |  |  |  |  |  |  |
|  | HLA-B*40:01 |  |  |  | 0.00004 | 28 |  |  |  |  |  |  |  |  |  |
|  | HLA-A*31:01 |  |  |  | 0.000025 | 54 |  |  |  |  |  |  |  |  |  |
|  | HLA-A*03:01 |  |  |  | 0.000024 | 47 |  |  |  |  |  |  |  |  |  |
|  | HLA-A*02:01 |  |  |  | 0.000022 | 54 |  |  |  |  |  |  |  |  |  |
|  | HLA-A*11:01 |  |  |  | 0.000019 | 38 |  |  |  |  |  |  |  |  |  |
| KFLPFQQFGR | HLA-A*31:01 | 558 | 567 | 10 | 0.786339 | 0.07 |  | 0.4 | Non-Allergen |  | 0.441 | Antigen |  | -0.84 | Non-toxic |
|  | HLA-A*33:01 |  |  |  | 0.218108 | 0.66 |  |  |  |  |  |  |  |  |  |
|  | HLA-A*30:01 |  |  |  | 0.087226 | 1.8 |  |  |  |  |  |  |  |  |  |
|  | HLA-A*03:01 |  |  |  | 0.035091 | 2.6 |  |  |  |  |  |  |  |  |  |
|  | HLA-A*68:01 |  |  |  | 0.030693 | 4.4 |  |  |  |  |  |  |  |  |  |
|  | HLA-A*30:02 |  |  |  | 0.014063 | 5.1 |  |  |  |  |  |  |  |  |  |
|  | HLA-A*11:01 |  |  |  | 0.013419 | 3.7 |  |  |  |  |  |  |  |  |  |
|  | HLA-A*23:01 |  |  |  | 0.002029 | 6.1 |  |  |  |  |  |  |  |  |  |
|  | HLA-A*24:02 |  |  |  | 0.001896 | 6.3 |  |  |  |  |  |  |  |  |  |
|  | HLA-A*32:01 |  |  |  | 0.001081 | 11 |  |  |  |  |  |  |  |  |  |
|  | HLA-B*57:01 |  |  |  | 0.000748 | 22 |  |  |  |  |  |  |  |  |  |
|  | HLA-B*58:01 |  |  |  | 0.000306 | 23 |  |  |  |  |  |  |  |  |  |
|  | HLA-A*01:01 |  |  |  | 0.000267 | 31 |  |  |  |  |  |  |  |  |  |
|  | HLA-A*02:01 |  |  |  | 0.000218 | 26 |  |  |  |  |  |  |  |  |  |
|  | HLA-A*02:06 |  |  |  | 0.000207 | 35 |  |  |  |  |  |  |  |  |  |
|  | HLA-A*26:01 |  |  |  | 0.000169 | 24 |  |  |  |  |  |  |  |  |  |
|  | HLA-B*08:01 |  |  |  | 0.000152 | 41 |  |  |  |  |  |  |  |  |  |
|  | HLA-A*02:03 |  |  |  | 0.000106 | 36 |  |  |  |  |  |  |  |  |  |
|  | HLA-A*68:02 |  |  |  | 0.000081 | 39 |  |  |  |  |  |  |  |  |  |
|  | HLA-B*15:01 |  |  |  | 0.000038 | 47 |  |  |  |  |  |  |  |  |  |
|  | HLA-B*44:02 |  |  |  | 0.000035 | 35 |  |  |  |  |  |  |  |  |  |
|  | HLA-B*53:01 |  |  |  | 0.000034 | 34 |  |  |  |  |  |  |  |  |  |
|  | HLA-B*44:03 |  |  |  | 0.000033 | 33 |  |  |  |  |  |  |  |  |  |
|  | HLA-B*40:01 |  |  |  | 0.00003 | 31 |  |  |  |  |  |  |  |  |  |
|  | HLA-B*51:01 |  |  |  | 0.000028 | 61 |  |  |  |  |  |  |  |  |  |
|  | HLA-B*07:02 |  |  |  | 0.000016 | 58 |  |  |  |  |  |  |  |  |  |
|  | HLA-B*35:01 |  |  |  | 0.000008 | 54 |  |  |  |  |  |  |  |  |  |
| KIYSKHTPI | HLA-A*32:01 | 202 | 210 | 9 | 0.553766 | 0.07 |  | 0.35 | Non-Allergen |  | 0.7455 | Antigen |  | -1.09 | Non-toxic |
|  | HLA-A*30:01 |  |  |  | 0.495841 | 0.14 |  |  |  |  |  |  |  |  |  |
|  | HLA-A*02:03 |  |  |  | 0.439809 | 0.29 |  |  |  |  |  |  |  |  |  |
|  | HLA-B*08:01 |  |  |  | 0.324637 | 0.32 |  |  |  |  |  |  |  |  |  |
|  | HLA-A*02:06 |  |  |  | 0.203623 | 0.86 |  |  |  |  |  |  |  |  |  |
|  | HLA-A*02:01 |  |  |  | 0.161841 | 0.97 |  |  |  |  |  |  |  |  |  |
|  | HLA-B*07:02 |  |  |  | 0.088574 | 1.3 |  |  |  |  |  |  |  |  |  |
|  | HLA-A*30:02 |  |  |  | 0.061021 | 1.9 |  |  |  |  |  |  |  |  |  |
|  | HLA-A*31:01 |  |  |  | 0.050056 | 3.1 |  |  |  |  |  |  |  |  |  |
|  | HLA-B*15:01 |  |  |  | 0.042138 | 2.7 |  |  |  |  |  |  |  |  |  |
|  | HLA-A*03:01 |  |  |  | 0.037826 | 2.5 |  |  |  |  |  |  |  |  |  |
|  | HLA-A*68:02 |  |  |  | 0.024021 | 3.1 |  |  |  |  |  |  |  |  |  |
|  | HLA-B*51:01 |  |  |  | 0.019029 | 4.2 |  |  |  |  |  |  |  |  |  |
|  | HLA-B*57:01 |  |  |  | 0.015418 | 5.1 |  |  |  |  |  |  |  |  |  |
|  | HLA-A*24:02 |  |  |  | 0.005034 | 4.1 |  |  |  |  |  |  |  |  |  |
|  | HLA-B*58:01 |  |  |  | 0.004519 | 6.3 |  |  |  |  |  |  |  |  |  |
|  | HLA-A*23:01 |  |  |  | 0.004475 | 4.3 |  |  |  |  |  |  |  |  |  |
|  | HLA-A*26:01 |  |  |  | 0.002932 | 6.4 |  |  |  |  |  |  |  |  |  |
|  | HLA-A*11:01 |  |  |  | 0.002684 | 6.8 |  |  |  |  |  |  |  |  |  |
|  | HLA-A*33:01 |  |  |  | 0.001506 | 12 |  |  |  |  |  |  |  |  |  |
|  | HLA-A*01:01 |  |  |  | 0.000785 | 17 |  |  |  |  |  |  |  |  |  |
|  | HLA-B*35:01 |  |  |  | 0.000747 | 12 |  |  |  |  |  |  |  |  |  |
|  | HLA-B*40:01 |  |  |  | 0.000477 | 11 |  |  |  |  |  |  |  |  |  |
|  | HLA-A*68:01 |  |  |  | 0.000429 | 20 |  |  |  |  |  |  |  |  |  |
|  | HLA-B*44:02 |  |  |  | 0.000325 | 14 |  |  |  |  |  |  |  |  |  |
|  | HLA-B*53:01 |  |  |  | 0.000294 | 16 |  |  |  |  |  |  |  |  |  |
|  | HLA-B*44:03 |  |  |  | 0.000176 | 17 |  |  |  |  |  |  |  |  |  |
| KQLSSKFGAI | HLA-A*02:06 | 964 | 973 | 10 | 0.043748 | 3 |  | 0.34 | Non-Allergen |  | 0.568 | Antigen |  | -0.54 | Non-toxic |
|  | HLA-A*32:01 |  |  |  | 0.0229 | 2.2 |  |  |  |  |  |  |  |  |  |
|  | HLA-A*02:03 |  |  |  | 0.022697 | 3.7 |  |  |  |  |  |  |  |  |  |
|  | HLA-A*30:01 |  |  |  | 0.010851 | 8 |  |  |  |  |  |  |  |  |  |
|  | HLA-B*15:01 |  |  |  | 0.009333 | 5.4 |  |  |  |  |  |  |  |  |  |
|  | HLA-A*02:01 |  |  |  | 0.006746 | 6 |  |  |  |  |  |  |  |  |  |
|  | HLA-A*30:02 |  |  |  | 0.005681 | 8.9 |  |  |  |  |  |  |  |  |  |
|  | HLA-B*08:01 |  |  |  | 0.002275 | 14 |  |  |  |  |  |  |  |  |  |
|  | HLA-B*57:01 |  |  |  | 0.001883 | 15 |  |  |  |  |  |  |  |  |  |
|  | HLA-A*24:02 |  |  |  | 0.001314 | 7.2 |  |  |  |  |  |  |  |  |  |
|  | HLA-B*40:01 |  |  |  | 0.001313 | 6.7 |  |  |  |  |  |  |  |  |  |
|  | HLA-A*31:01 |  |  |  | 0.001269 | 15 |  |  |  |  |  |  |  |  |  |
|  | HLA-A*23:01 |  |  |  | 0.000972 | 8.3 |  |  |  |  |  |  |  |  |  |
|  | HLA-B*58:01 |  |  |  | 0.000911 | 14 |  |  |  |  |  |  |  |  |  |
|  | HLA-B*07:02 |  |  |  | 0.00052 | 16 |  |  |  |  |  |  |  |  |  |
|  | HLA-B*51:01 |  |  |  | 0.000495 | 24 |  |  |  |  |  |  |  |  |  |
|  | HLA-B*44:03 |  |  |  | 0.000381 | 12 |  |  |  |  |  |  |  |  |  |
|  | HLA-B*44:02 |  |  |  | 0.000306 | 14 |  |  |  |  |  |  |  |  |  |
|  | HLA-A*03:01 |  |  |  | 0.000269 | 20 |  |  |  |  |  |  |  |  |  |
|  | HLA-A*68:02 |  |  |  | 0.000266 | 26 |  |  |  |  |  |  |  |  |  |
|  | HLA-A*01:01 |  |  |  | 0.000123 | 45 |  |  |  |  |  |  |  |  |  |
|  | HLA-A*26:01 |  |  |  | 0.000077 | 32 |  |  |  |  |  |  |  |  |  |
|  | HLA-A*33:01 |  |  |  | 0.000064 | 42 |  |  |  |  |  |  |  |  |  |
|  | HLA-A*11:01 |  |  |  | 0.000052 | 27 |  |  |  |  |  |  |  |  |  |
|  | HLA-B*53:01 |  |  |  | 0.000034 | 34 |  |  |  |  |  |  |  |  |  |
|  | HLA-B*35:01 |  |  |  | 0.000014 | 46 |  |  |  |  |  |  |  |  |  |
|  | HLA-A*68:01 |  |  |  | 0.000009 | 63 |  |  |  |  |  |  |  |  |  |
| LIDLQELGKY | HLA-A*01:01 | 1197 | 1206 | 10 | 0.791918 | 0.07 |  | 0.3 | Non-Allergen |  | 0.7076 | Antigen |  | -0.91 | Non-toxic |
|  | HLA-A*30:02 |  |  |  | 0.116619 | 1.2 |  |  |  |  |  |  |  |  |  |
|  | HLA-B*15:01 |  |  |  | 0.049902 | 2.5 |  |  |  |  |  |  |  |  |  |
|  | HLA-A*26:01 |  |  |  | 0.016617 | 2.4 |  |  |  |  |  |  |  |  |  |
|  | HLA-B*35:01 |  |  |  | 0.00619 | 4.6 |  |  |  |  |  |  |  |  |  |
|  | HLA-B*57:01 |  |  |  | 0.004544 | 9.3 |  |  |  |  |  |  |  |  |  |
|  | HLA-A*03:01 |  |  |  | 0.002629 | 8 |  |  |  |  |  |  |  |  |  |
|  | HLA-B*58:01 |  |  |  | 0.002175 | 9.1 |  |  |  |  |  |  |  |  |  |
|  | HLA-B*53:01 |  |  |  | 0.00201 | 6.8 |  |  |  |  |  |  |  |  |  |
|  | HLA-A*11:01 |  |  |  | 0.001998 | 7.5 |  |  |  |  |  |  |  |  |  |
|  | HLA-B*44:03 |  |  |  | 0.001046 | 7.8 |  |  |  |  |  |  |  |  |  |
|  | HLA-A*68:01 |  |  |  | 0.00104 | 15 |  |  |  |  |  |  |  |  |  |
|  | HLA-B*44:02 |  |  |  | 0.00075 | 9.1 |  |  |  |  |  |  |  |  |  |
|  | HLA-A*32:01 |  |  |  | 0.000719 | 12 |  |  |  |  |  |  |  |  |  |
|  | HLA-A*02:06 |  |  |  | 0.000443 | 27 |  |  |  |  |  |  |  |  |  |
|  | HLA-A*02:01 |  |  |  | 0.000324 | 22 |  |  |  |  |  |  |  |  |  |
|  | HLA-A*33:01 |  |  |  | 0.000263 | 24 |  |  |  |  |  |  |  |  |  |
|  | HLA-A*31:01 |  |  |  | 0.000242 | 26 |  |  |  |  |  |  |  |  |  |
|  | HLA-B*51:01 |  |  |  | 0.000151 | 37 |  |  |  |  |  |  |  |  |  |
|  | HLA-B*07:02 |  |  |  | 0.000113 | 30 |  |  |  |  |  |  |  |  |  |
|  | HLA-B*08:01 |  |  |  | 0.000112 | 46 |  |  |  |  |  |  |  |  |  |
|  | HLA-A*02:03 |  |  |  | 0.000109 | 36 |  |  |  |  |  |  |  |  |  |
|  | HLA-A*30:01 |  |  |  | 0.000108 | 58 |  |  |  |  |  |  |  |  |  |
|  | HLA-A*24:02 |  |  |  | 0.000029 | 32 |  |  |  |  |  |  |  |  |  |
|  | HLA-A*23:01 |  |  |  | 0.000028 | 34 |  |  |  |  |  |  |  |  |  |
|  | HLA-A*68:02 |  |  |  | 0.000019 | 57 |  |  |  |  |  |  |  |  |  |
|  | HLA-B*40:01 |  |  |  | 0.000019 | 35 |  |  |  |  |  |  |  |  |  |
| LPIGINITRF | HLA-B*35:01 | 229 | 238 | 10 | 0.828081 | 0.07 |  | 0.38 | Non-Allergen |  | 1.3027 | Antigen |  | -1.04 | Non-toxic |
|  | HLA-B*53:01 |  |  |  | 0.811192 | 0.03 |  |  |  |  |  |  |  |  |  |
|  | HLA-B*51:01 |  |  |  | 0.329148 | 0.43 |  |  |  |  |  |  |  |  |  |
|  | HLA-B*07:02 |  |  |  | 0.190649 | 0.71 |  |  |  |  |  |  |  |  |  |
|  | HLA-A*26:01 |  |  |  | 0.032192 | 1.6 |  |  |  |  |  |  |  |  |  |
|  | HLA-B*57:01 |  |  |  | 0.020774 | 4.5 |  |  |  |  |  |  |  |  |  |
|  | HLA-A*68:01 |  |  |  | 0.019172 | 5.4 |  |  |  |  |  |  |  |  |  |
|  | HLA-B*58:01 |  |  |  | 0.013637 | 3.6 |  |  |  |  |  |  |  |  |  |
|  | HLA-B*08:01 |  |  |  | 0.012716 | 5.2 |  |  |  |  |  |  |  |  |  |
|  | HLA-B*15:01 |  |  |  | 0.008623 | 5.6 |  |  |  |  |  |  |  |  |  |
|  | HLA-A*23:01 |  |  |  | 0.008238 | 3.2 |  |  |  |  |  |  |  |  |  |
|  | HLA-A*24:02 |  |  |  | 0.004563 | 4.3 |  |  |  |  |  |  |  |  |  |
|  | HLA-A*68:02 |  |  |  | 0.003711 | 8.3 |  |  |  |  |  |  |  |  |  |
|  | HLA-A*33:01 |  |  |  | 0.003241 | 8 |  |  |  |  |  |  |  |  |  |
|  | HLA-A*32:01 |  |  |  | 0.002013 | 7.6 |  |  |  |  |  |  |  |  |  |
|  | HLA-A*30:02 |  |  |  | 0.001977 | 17 |  |  |  |  |  |  |  |  |  |
|  | HLA-A*01:01 |  |  |  | 0.001541 | 12 |  |  |  |  |  |  |  |  |  |
|  | HLA-B*44:03 |  |  |  | 0.001124 | 7.6 |  |  |  |  |  |  |  |  |  |
|  | HLA-B*44:02 |  |  |  | 0.00088 | 8.4 |  |  |  |  |  |  |  |  |  |
|  | HLA-A*02:06 |  |  |  | 0.000599 | 24 |  |  |  |  |  |  |  |  |  |
|  | HLA-A*31:01 |  |  |  | 0.0004 | 22 |  |  |  |  |  |  |  |  |  |
|  | HLA-B*40:01 |  |  |  | 0.000318 | 13 |  |  |  |  |  |  |  |  |  |
|  | HLA-A*03:01 |  |  |  | 0.000227 | 21 |  |  |  |  |  |  |  |  |  |
|  | HLA-A*30:01 |  |  |  | 0.000182 | 50 |  |  |  |  |  |  |  |  |  |
|  | HLA-A*02:01 |  |  |  | 0.00018 | 28 |  |  |  |  |  |  |  |  |  |
|  | HLA-A*02:03 |  |  |  | 0.000142 | 33 |  |  |  |  |  |  |  |  |  |
|  | HLA-A*11:01 |  |  |  | 0.000096 | 22 |  |  |  |  |  |  |  |  |  |
| RQIAPGQTGK | HLA-A*03:01 | 408 | 417 | 10 | 0.904489 | 0.03 |  | 0.35 | Non-Allergen |  | 1.7893 | Antigen |  | -0.79 | Non-toxic |
|  | HLA-A*11:01 |  |  |  | 0.715172 | 0.13 |  |  |  |  |  |  |  |  |  |
|  | HLA-A*30:01 |  |  |  | 0.473041 | 0.16 |  |  |  |  |  |  |  |  |  |
|  | HLA-A*31:01 |  |  |  | 0.150179 | 1.5 |  |  |  |  |  |  |  |  |  |
|  | HLA-A*68:01 |  |  |  | 0.078354 | 2.9 |  |  |  |  |  |  |  |  |  |
|  | HLA-A*30:02 |  |  |  | 0.0768 | 1.7 |  |  |  |  |  |  |  |  |  |
|  | HLA-B*15:01 |  |  |  | 0.04994 | 2.5 |  |  |  |  |  |  |  |  |  |
|  | HLA-A*32:01 |  |  |  | 0.013042 | 3 |  |  |  |  |  |  |  |  |  |
|  | HLA-A*02:06 |  |  |  | 0.012388 | 6.2 |  |  |  |  |  |  |  |  |  |
|  | HLA-B*57:01 |  |  |  | 0.003576 | 11 |  |  |  |  |  |  |  |  |  |
|  | HLA-A*02:03 |  |  |  | 0.003535 | 9.2 |  |  |  |  |  |  |  |  |  |
|  | HLA-A*02:01 |  |  |  | 0.002282 | 9.7 |  |  |  |  |  |  |  |  |  |
|  | HLA-B*58:01 |  |  |  | 0.00226 | 9 |  |  |  |  |  |  |  |  |  |
|  | HLA-B*40:01 |  |  |  | 0.002127 | 5.4 |  |  |  |  |  |  |  |  |  |
|  | HLA-B*44:03 |  |  |  | 0.002048 | 5.9 |  |  |  |  |  |  |  |  |  |
|  | HLA-B*44:02 |  |  |  | 0.001871 | 6 |  |  |  |  |  |  |  |  |  |
|  | HLA-A*33:01 |  |  |  | 0.001539 | 12 |  |  |  |  |  |  |  |  |  |
|  | HLA-A*26:01 |  |  |  | 0.001466 | 9 |  |  |  |  |  |  |  |  |  |
|  | HLA-A*01:01 |  |  |  | 0.001455 | 12 |  |  |  |  |  |  |  |  |  |
|  | HLA-B*07:02 |  |  |  | 0.000996 | 12 |  |  |  |  |  |  |  |  |  |
|  | HLA-A*23:01 |  |  |  | 0.000344 | 13 |  |  |  |  |  |  |  |  |  |
|  | HLA-A*68:02 |  |  |  | 0.000281 | 26 |  |  |  |  |  |  |  |  |  |
|  | HLA-A*24:02 |  |  |  | 0.000239 | 15 |  |  |  |  |  |  |  |  |  |
|  | HLA-B*35:01 |  |  |  | 0.00014 | 22 |  |  |  |  |  |  |  |  |  |
|  | HLA-B*08:01 |  |  |  | 0.000131 | 44 |  |  |  |  |  |  |  |  |  |
|  | HLA-B*51:01 |  |  |  | 0.000127 | 39 |  |  |  |  |  |  |  |  |  |
|  | HLA-B*53:01 |  |  |  | 0.000059 | 28 |  |  |  |  |  |  |  |  |  |
| CYFPLRSYSF | HLA-A*24:02 | 488 | 497 | 10 | 0.730103 | 0.08 |  | 0.32 | Non-Allergen |  | 1.5062 | Antigen |  | -0.58 | Non-toxic |
|  | HLA-A*23:01 |  |  |  | 0.729049 | 0.07 |  |  |  |  |  |  |  |  |  |
|  | HLA-A*30:02 |  |  |  | 0.029912 | 3.2 |  |  |  |  |  |  |  |  |  |
|  | HLA-B*57:01 |  |  |  | 0.029514 | 3.8 |  |  |  |  |  |  |  |  |  |
|  | HLA-A*32:01 |  |  |  | 0.02315 | 2.2 |  |  |  |  |  |  |  |  |  |
|  | HLA-B*08:01 |  |  |  | 0.020333 | 3.9 |  |  |  |  |  |  |  |  |  |
|  | HLA-A*33:01 |  |  |  | 0.01957 | 3.7 |  |  |  |  |  |  |  |  |  |
|  | HLA-A*31:01 |  |  |  | 0.009617 | 6.5 |  |  |  |  |  |  |  |  |  |
|  | HLA-B*58:01 |  |  |  | 0.005848 | 5.5 |  |  |  |  |  |  |  |  |  |
|  | HLA-A*30:01 |  |  |  | 0.005768 | 12 |  |  |  |  |  |  |  |  |  |
|  | HLA-B*15:01 |  |  |  | 0.005494 | 6.8 |  |  |  |  |  |  |  |  |  |
|  | HLA-B*53:01 |  |  |  | 0.004992 | 4.6 |  |  |  |  |  |  |  |  |  |
|  | HLA-A*26:01 |  |  |  | 0.002999 | 6.3 |  |  |  |  |  |  |  |  |  |
|  | HLA-B*35:01 |  |  |  | 0.002485 | 6.9 |  |  |  |  |  |  |  |  |  |
|  | HLA-B*51:01 |  |  |  | 0.001193 | 17 |  |  |  |  |  |  |  |  |  |
|  | HLA-B*07:02 |  |  |  | 0.001063 | 12 |  |  |  |  |  |  |  |  |  |
|  | HLA-A*68:01 |  |  |  | 0.000825 | 16 |  |  |  |  |  |  |  |  |  |
|  | HLA-A*01:01 |  |  |  | 0.000758 | 18 |  |  |  |  |  |  |  |  |  |
|  | HLA-B*44:02 |  |  |  | 0.000637 | 9.8 |  |  |  |  |  |  |  |  |  |
|  | HLA-B*44:03 |  |  |  | 0.000603 | 9.9 |  |  |  |  |  |  |  |  |  |
|  | HLA-A*02:06 |  |  |  | 0.000313 | 30 |  |  |  |  |  |  |  |  |  |
|  | HLA-A*03:01 |  |  |  | 0.000201 | 22 |  |  |  |  |  |  |  |  |  |
|  | HLA-A*68:02 |  |  |  | 0.000161 | 31 |  |  |  |  |  |  |  |  |  |
|  | HLA-A*11:01 |  |  |  | 0.00011 | 21 |  |  |  |  |  |  |  |  |  |
|  | HLA-B*40:01 |  |  |  | 0.000107 | 19 |  |  |  |  |  |  |  |  |  |
|  | HLA-A*02:03 |  |  |  | 0.000076 | 40 |  |  |  |  |  |  |  |  |  |
|  | HLA-A*02:01 |  |  |  | 0.000072 | 38 |  |  |  |  |  |  |  |  |  |
| DISGINASV | HLA-A*68:02 | 1168 | 1176 | 9 | 0.887294 | 0.02 |  | 0.32 | Non-Allergen |  | 0.4155 | Antigen |  | -0.94 | Non-toxic |
|  | HLA-A*26:01 |  |  |  | 0.128976 | 0.55 |  |  |  |  |  |  |  |  |  |
|  | HLA-B*51:01 |  |  |  | 0.10192 | 1.3 |  |  |  |  |  |  |  |  |  |
|  | HLA-A*02:03 |  |  |  | 0.054675 | 2.2 |  |  |  |  |  |  |  |  |  |
|  | HLA-A*02:06 |  |  |  | 0.046411 | 2.8 |  |  |  |  |  |  |  |  |  |
|  | HLA-B*08:01 |  |  |  | 0.019655 | 4 |  |  |  |  |  |  |  |  |  |
|  | HLA-A*02:01 |  |  |  | 0.019632 | 3.6 |  |  |  |  |  |  |  |  |  |
|  | HLA-A*68:01 |  |  |  | 0.017062 | 5.6 |  |  |  |  |  |  |  |  |  |
|  | HLA-B*35:01 |  |  |  | 0.009762 | 3.8 |  |  |  |  |  |  |  |  |  |
|  | HLA-A*33:01 |  |  |  | 0.007871 | 5.6 |  |  |  |  |  |  |  |  |  |
|  | HLA-B*53:01 |  |  |  | 0.003822 | 5.2 |  |  |  |  |  |  |  |  |  |
|  | HLA-A*30:02 |  |  |  | 0.002278 | 16 |  |  |  |  |  |  |  |  |  |
|  | HLA-B*07:02 |  |  |  | 0.002187 | 8.1 |  |  |  |  |  |  |  |  |  |
|  | HLA-A*01:01 |  |  |  | 0.001585 | 12 |  |  |  |  |  |  |  |  |  |
|  | HLA-A*30:01 |  |  |  | 0.001375 | 23 |  |  |  |  |  |  |  |  |  |
|  | HLA-A*32:01 |  |  |  | 0.000556 | 14 |  |  |  |  |  |  |  |  |  |
|  | HLA-B*15:01 |  |  |  | 0.000477 | 19 |  |  |  |  |  |  |  |  |  |
|  | HLA-A*31:01 |  |  |  | 0.000431 | 22 |  |  |  |  |  |  |  |  |  |
|  | HLA-B*57:01 |  |  |  | 0.000328 | 31 |  |  |  |  |  |  |  |  |  |
|  | HLA-B*44:03 |  |  |  | 0.000307 | 14 |  |  |  |  |  |  |  |  |  |
|  | HLA-B*44:02 |  |  |  | 0.00029 | 14 |  |  |  |  |  |  |  |  |  |
|  | HLA-B*40:01 |  |  |  | 0.000181 | 16 |  |  |  |  |  |  |  |  |  |
|  | HLA-A*03:01 |  |  |  | 0.000171 | 23 |  |  |  |  |  |  |  |  |  |
|  | HLA-B*58:01 |  |  |  | 0.000157 | 31 |  |  |  |  |  |  |  |  |  |
|  | HLA-A*23:01 |  |  |  | 0.000115 | 20 |  |  |  |  |  |  |  |  |  |
|  | HLA-A*24:02 |  |  |  | 0.000068 | 24 |  |  |  |  |  |  |  |  |  |
|  | HLA-A*11:01 |  |  |  | 0.000066 | 25 |  |  |  |  |  |  |  |  |  |
| **MHC II** | | | | | | | | | | | | | | | |
| **IEDB** | | | | | | |  | **Algpred** | |  | **Vaxijen** | |  | **ToxinPred** | |
| **Epitope** | **MHC allele** | **Start** | **End** | **Length** | **Percentile rank** | **Adjusted rank** |  | **Score** | **Prediction** |  | **Score** | **Prediction** |  | **SVM score** | **Prediction** |
| ASYQTQTKSHRRARS | HLA-DRB3*02:02 | 672 | 686 | 15 | 46 | 46 |  | 0.3 | Non-Allergen |  | 0.7544 | Antigen |  | -1.24 | Non-toxic |
| CTQLKRALTGIAVEQ | HLA-DRB3*02:02 | 760 | 774 | 15 | 21 | 12 |  | 0.35 | Non-Allergen |  | 0.7763 | Antigen |  | -1.38 | Non-toxic |
| GDEVRQIAPGQTGKI | HLA-DRB3*02:02 | 404 | 418 | 15 | 65 | 65 |  | 0.37 | Non-Allergen |  | 0.9741 | Antigen |  | -0.76 | Non-toxic |
| SAIGKIQDSLSST | HLA-DRB3*02:02 | 929 | 941 | 13 | 58 | 90.44 |  | 0.35 | Non-Allergen |  | 0.5434 | Antigen |  | -1.28 | Non-toxic |
| SECVLGQSKRVDFCGKGYHL | HLA-DRB3*02:02 | 1030 | 1049 | 20 | 72 | 556.4 |  | 0.34 | Non-Allergen |  | 0.9179 | Antigen |  | -0.09 | Non-toxic |
| LDPLSETKCTLKSFT | HLA-DRB3*02:02 | 293 | 307 | 15 | 90 | 90 |  | 0.37 | Non-Allergen |  | 0.7712 | Antigen |  | -1.28 | Non-toxic |
| TISVTTEILPVSMT | HLA-DRB3*02:02 | 719 | 732 | 14 | 63 | 67.4 |  | 0.26 | Non-Allergen |  | 1.2621 | Antigen |  | -1.34 | Non-toxic |
| FPLRSYSFRPTYGVG | HLA-DRB3*02:02 | 490 | 504 | 15 | 39 | 39 |  | 0.32 | Non-Allergen |  | 0.7464 | Antigen |  | -1.13 | Non-toxic |
| DCLGDIAARDLI | HLA-DRB3*02:02 | 839 | 850 | 12 | 39 | 115.65 |  | 0.36 | Non-Allergen |  | 0.4525 | Antigen |  | -0.71 | Non-toxic |

**Supplementary information 3.** Selected B cell epitopes (ABCpred) used in this study.

| **BA.1** | | | | | | | | | | | |
| --- | --- | --- | --- | --- | --- | --- | --- | --- | --- | --- | --- |
| **B cell epitope** | | | | | | | | | | | |
| **ABCpred** | | |  | **AlgPred** | |  | **Vaxijen** | |  | **ToxinPred** | |
| **Epitope** | **Start position** | **Score** |  | **Score** | **Prediction** |  | **Score** | **Prediction** |  | **SVM score** | **Prediction** |
| GVSVITPGTNTSNQVA | 594 | 0.95 |  | 0.38 | Non-Allergen |  | 0.4651 | Antigen |  | -1.58 | Non-toxic |
| LRSYSFRPTYGVGHQP | 492 | 0.94 |  | 0.3 | Non-Allergen |  | 0.4532 | Antigen |  | -1.38 | Non-toxic |
| HRSYLTPGDSSSGWTA | 245 | 0.92 |  | 0.34 | Non-Allergen |  | 0.6017 | Antigen |  | -0.7 | Non-toxic |
| TRFQTLLALHRSYLTP | 236 | 0.89 |  | 0.32 | Non-Allergen |  | 0.5115 | Antigen |  | -0.98 | Non-toxic |
| YEQYIKWPWYIWLGFI | 1206 | 0.89 |  | 0.34 | Non-Allergen |  | 0.951 | Antigen |  | -0.35 | Non-toxic |

**Supplementary information 4.** Estimated physicochemical properties of all vaccine constructs.

| **#** | **Properties** |  | **Vaxign2** | |  | **IEDB** | |
| --- | --- | --- | --- | --- | --- | --- | --- |
|  |  |  | **BA. 1** | **BA.2** |  | **BA. 1** | **BA.2** |
| **1** | **Number of amino acids** |  | **567** | **569** |  | **562** | **564** |
| **2** | **Molecular weight** |  | **60 kDa** | **59.9 kDa** |  | **59.5 kDa** | **59.4 kDa** |
| **3** | **Acidic amino acids** |  | **67** | **66** |  | **66** | **65** |
| **4** | **Basic amino acids** |  | **77** | **73** |  | **76** | **72** |
| **5** | **Chemical formula** |  | **C_2676_H_4296_N_726_O_814_S_11_** | **C_2674_H_4281_N_725_O_816_S_11_** |  | **C_2654_H_4258_N_720_O_807_S_10_** | **C_2652_H_4243_N_719_O_809_S_10_** |
| **6** | **Estimated half-life in E. coli** |  | **> 10 h** | **> 10 h** |  | **> 10 h** | **> 10 h** |
| **7** | **Estimated half-life in mammalian cells** |  | **30 h** | **30 h** |  | **30 h** | **30 h** |
| **8** | **Estimated half-life in yeast** |  | **> 20 h** | **> 20 h** |  | **> 20 h** | **> 20 h** |
| **9** | **Instability index (II)** |  | **35.24** | **35.12** |  | **35.66** | **35.53** |
| **10** | **Aliphatic index** |  | **81.55** | **81.78** |  | **81.58** | **81.81** |
| **11** | **GRAVY** |  | **-0.163** | **-0.144** |  | **-0.166** | **-0.147** |
| **12** | **Solubility upon overexpression** |  | **0.95** | **0.96** |  | **0.94** | **0.95** |
| **13** | **Alpha-helix (H)** |  | **50%** | **45%** |  | **51%** | **46%** |
| **14** | **Beta-sheet (E)** |  | **6%** | **10%** |  | **5%** | **9%** |
| **15** | **Coil (C)** |  | **43%** | **44%** |  | **42%** | **44%** |

**Supplementary information** **5.** The top models produced by each server. Galaxy Refine was used to relieve the clashes if any in the models.

| **Vaxign 2** | | |
| --- | --- | --- |
| **Model** | **BA. 1** | **BA. 2** |
| AlphaFold 2 | 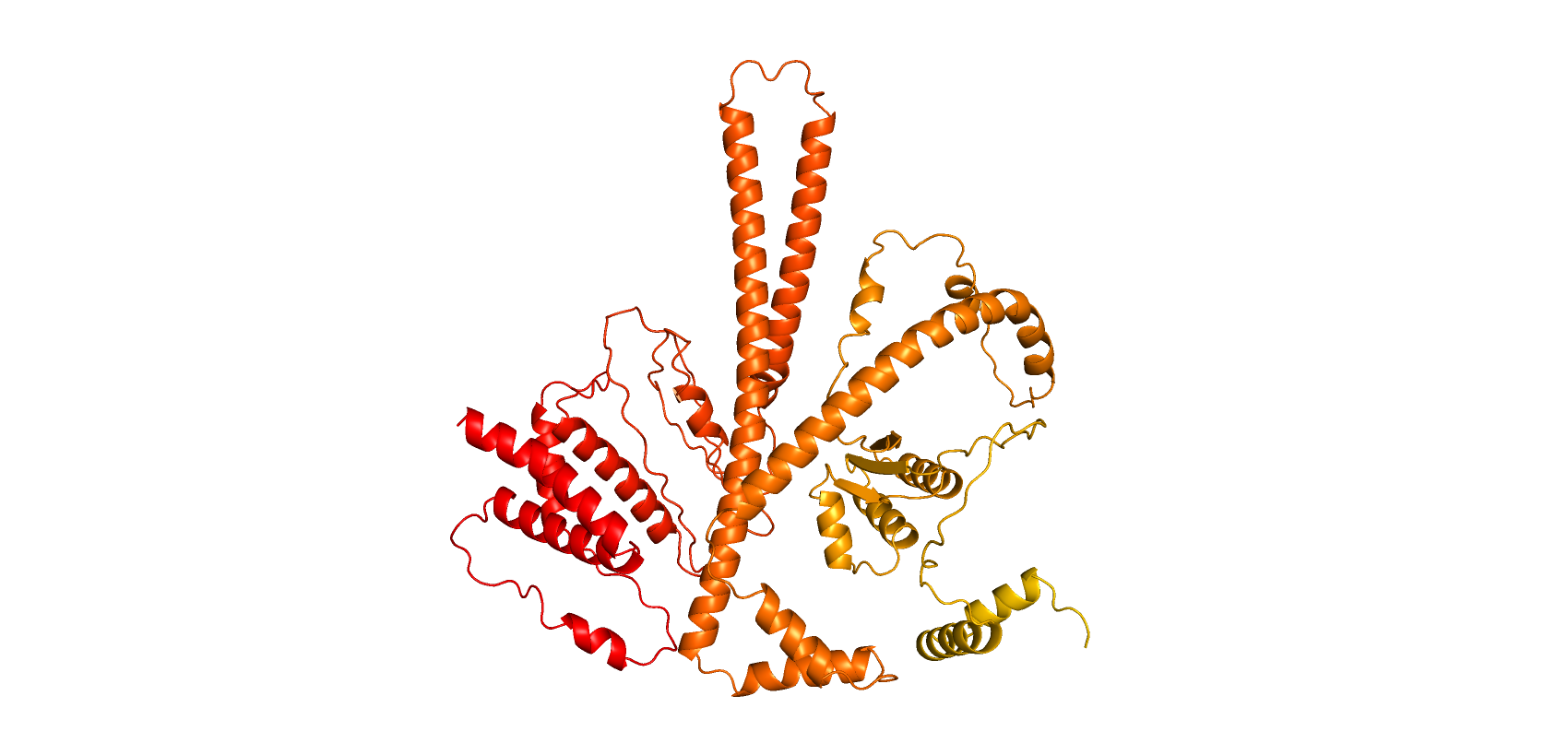 | 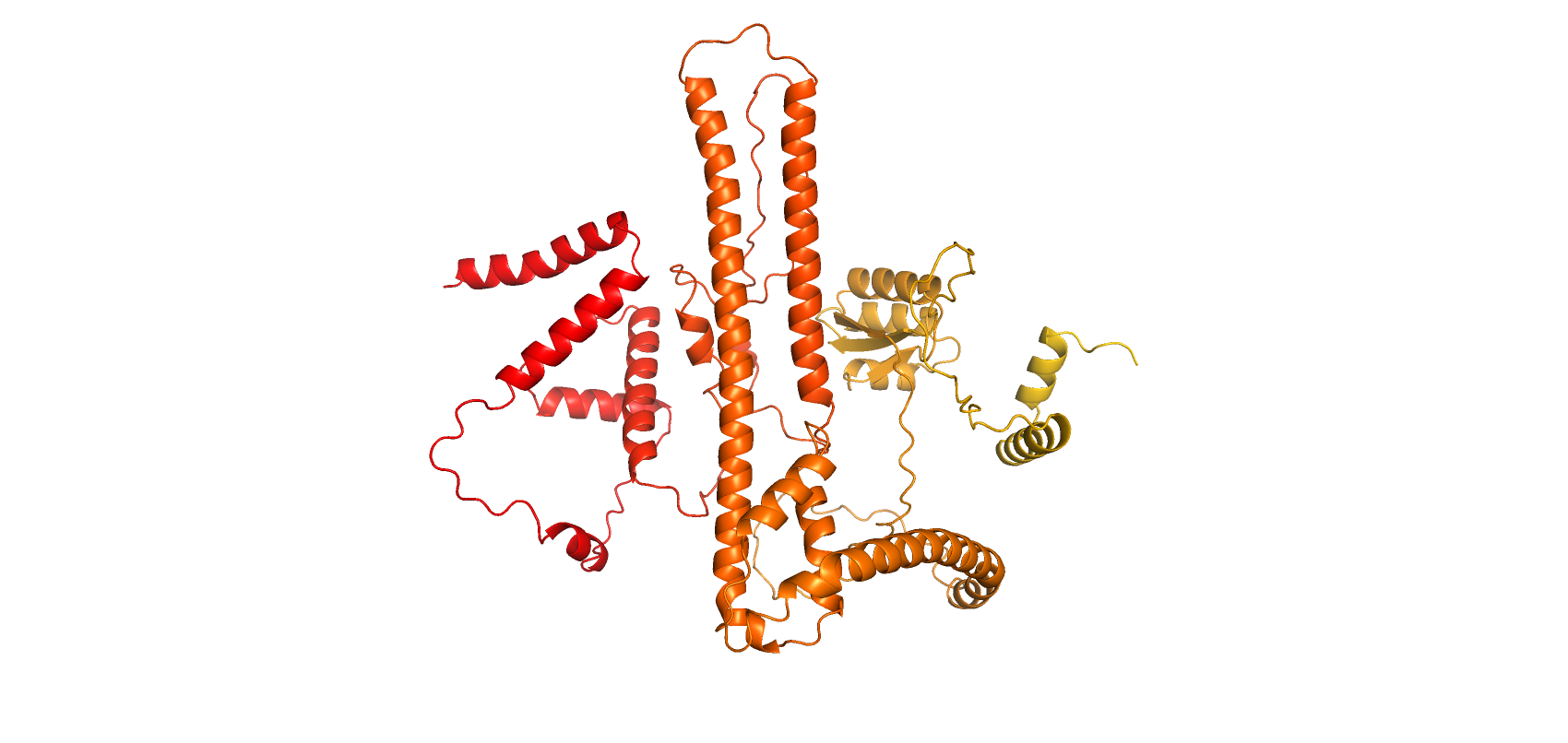 |
| RaptorX | 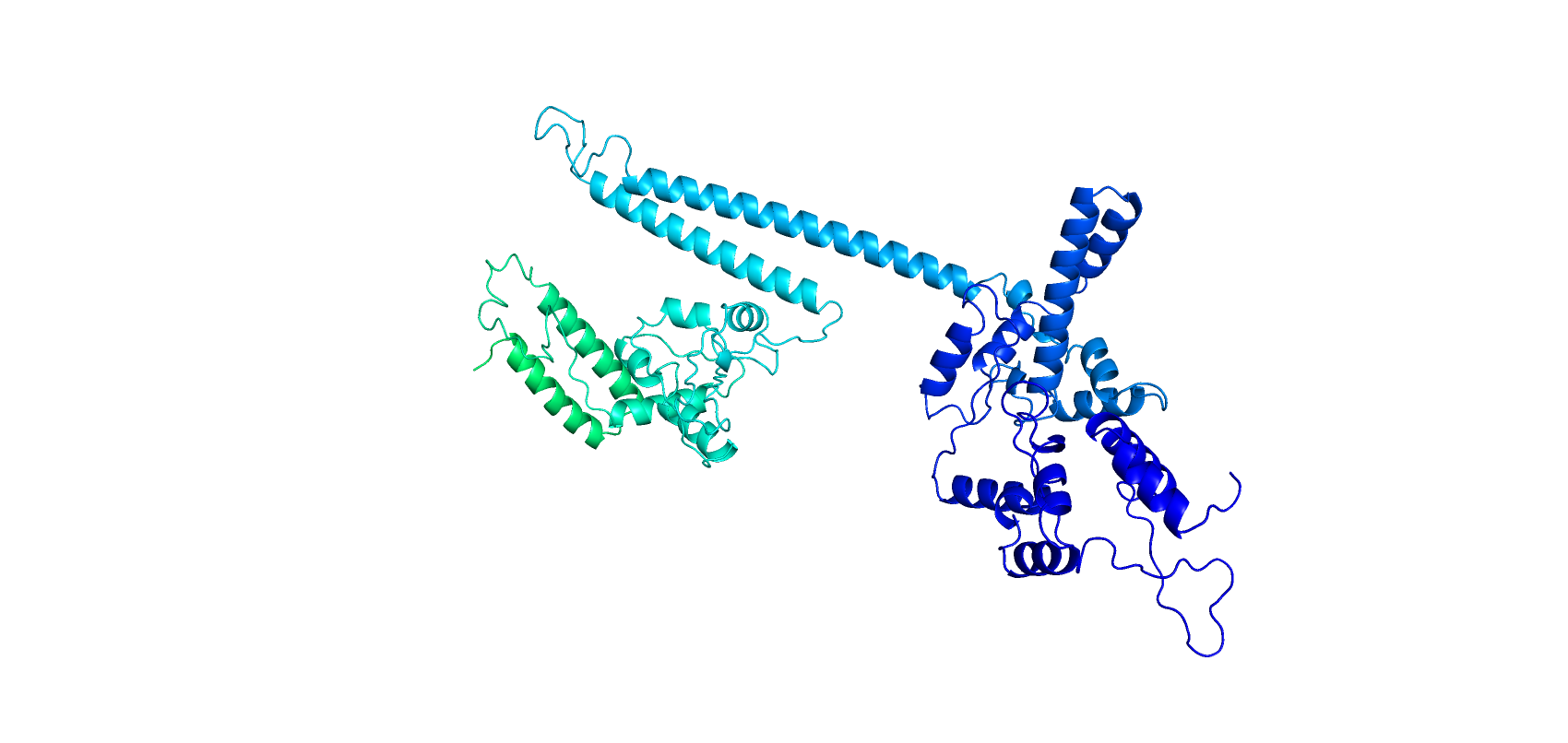 | 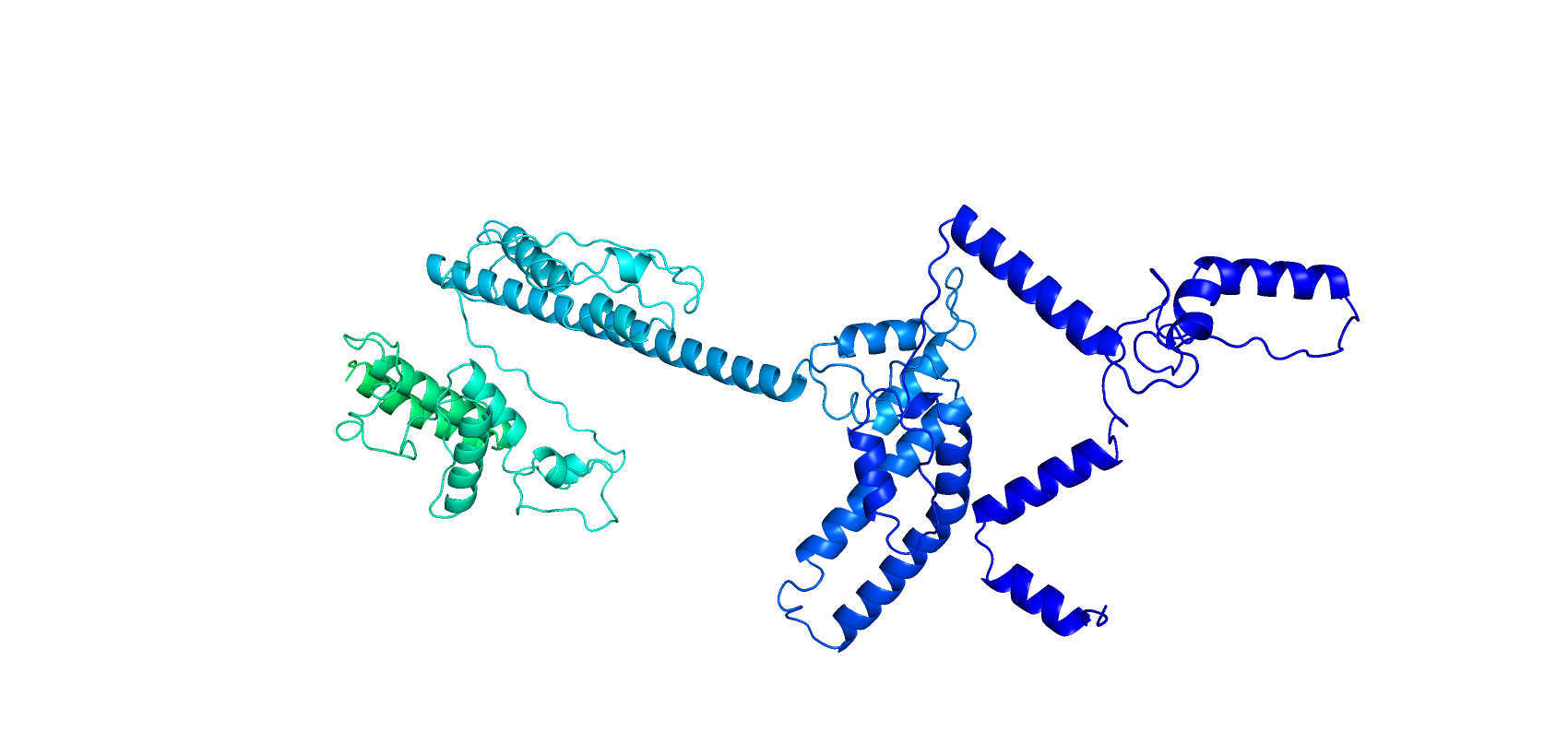 |
| RoseTTAfold | 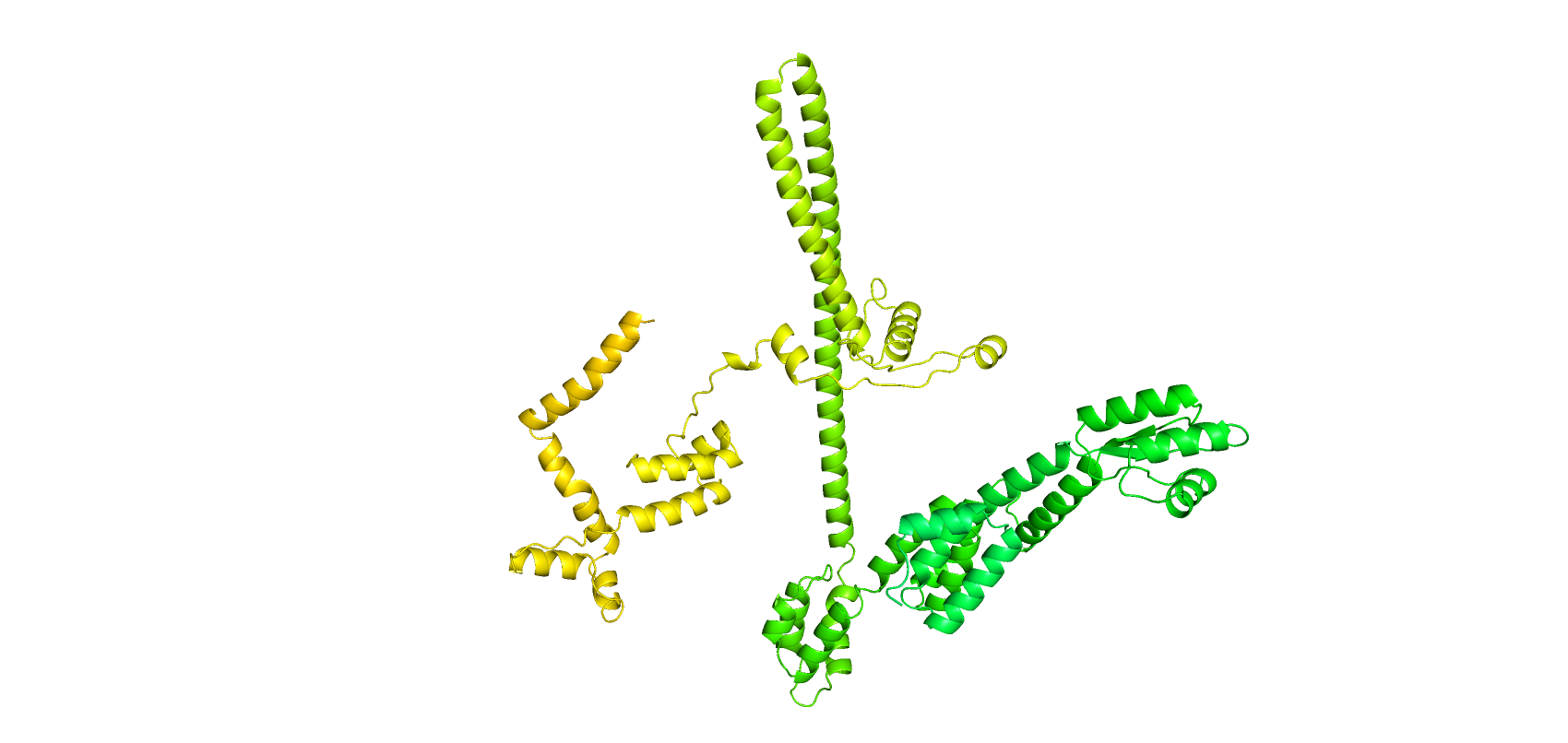 | 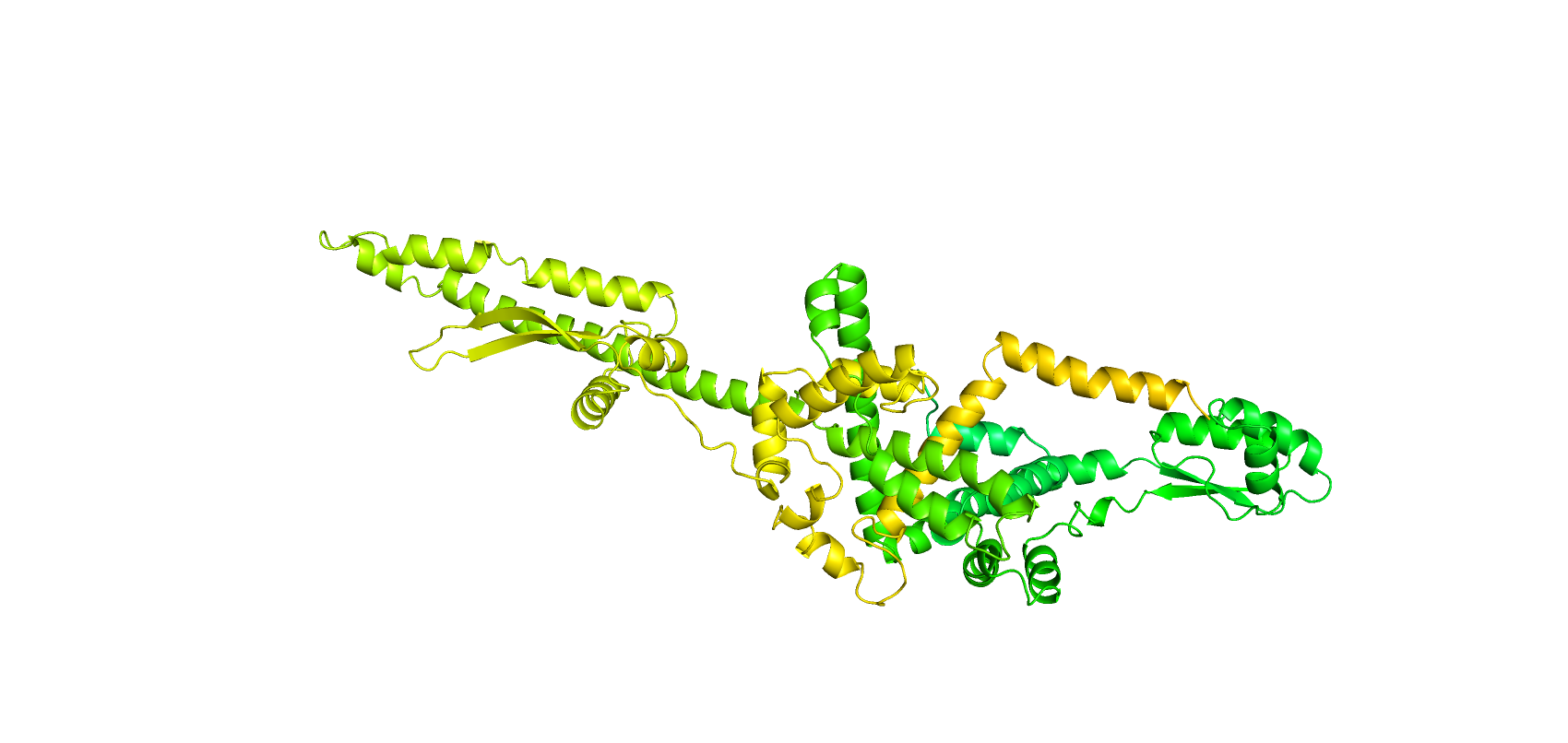 |
| **IEDB** | | |
| **Model** | **BA. 1** | **BA. 2** |
| AlphaFold 2 | 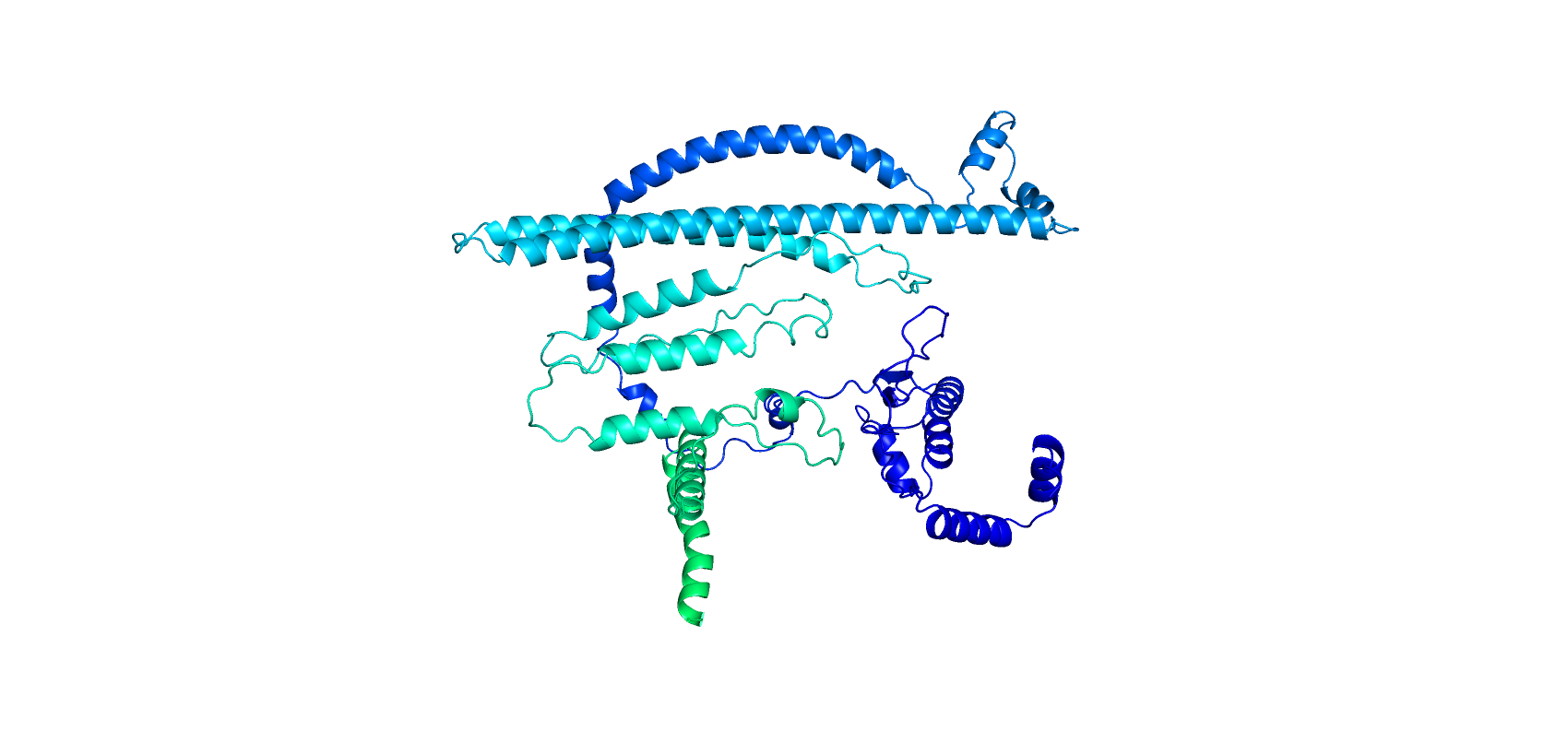 | 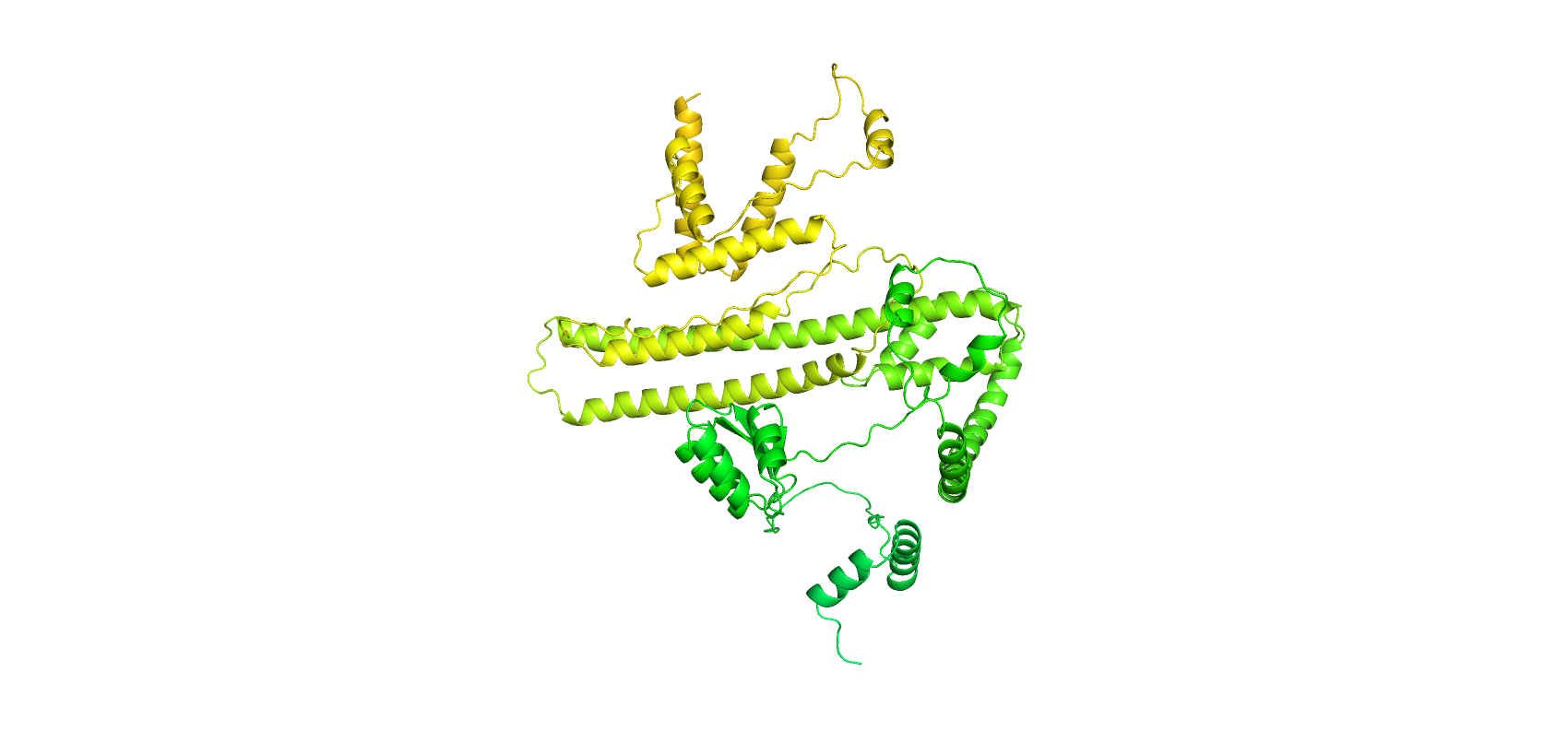 |
| RaptorX | 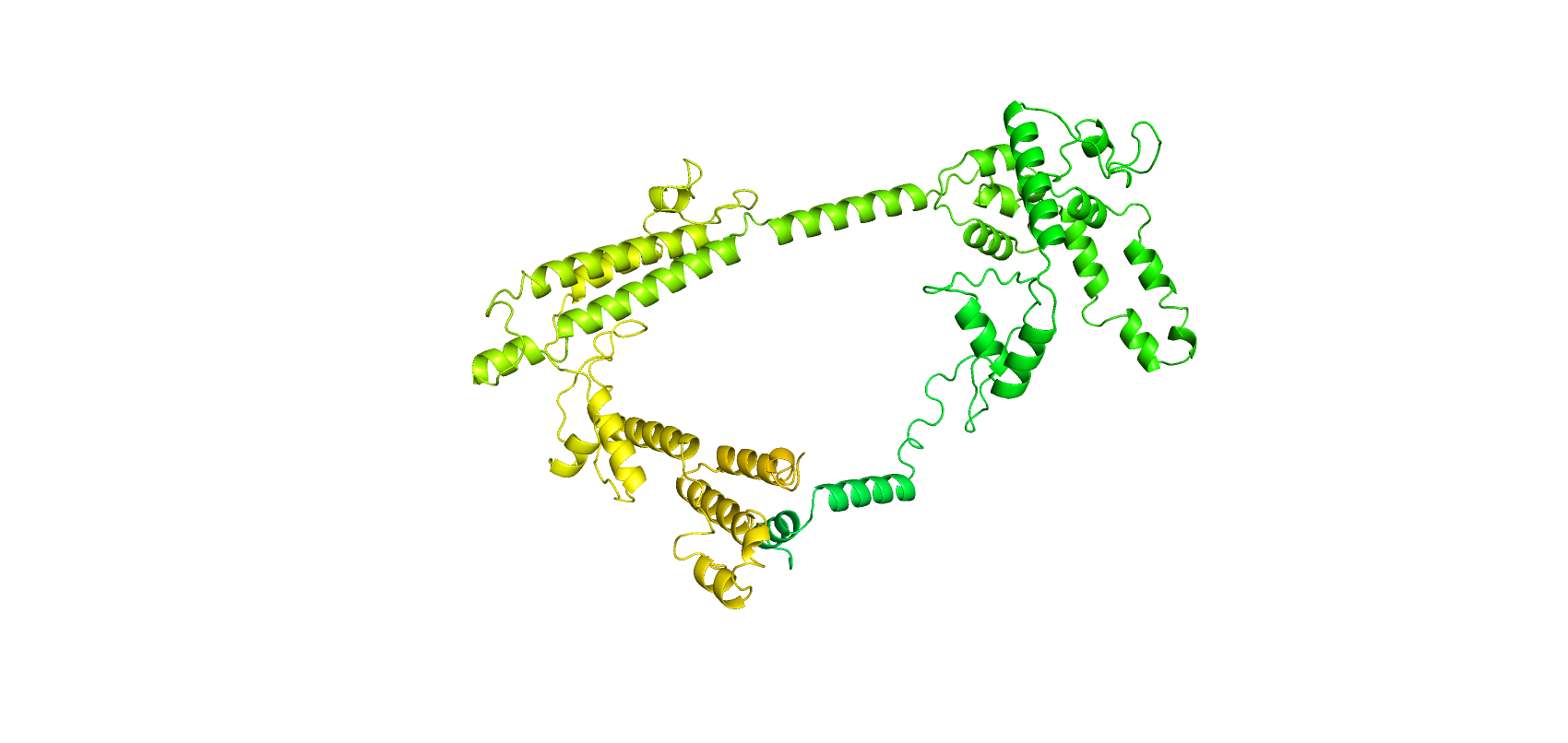 | 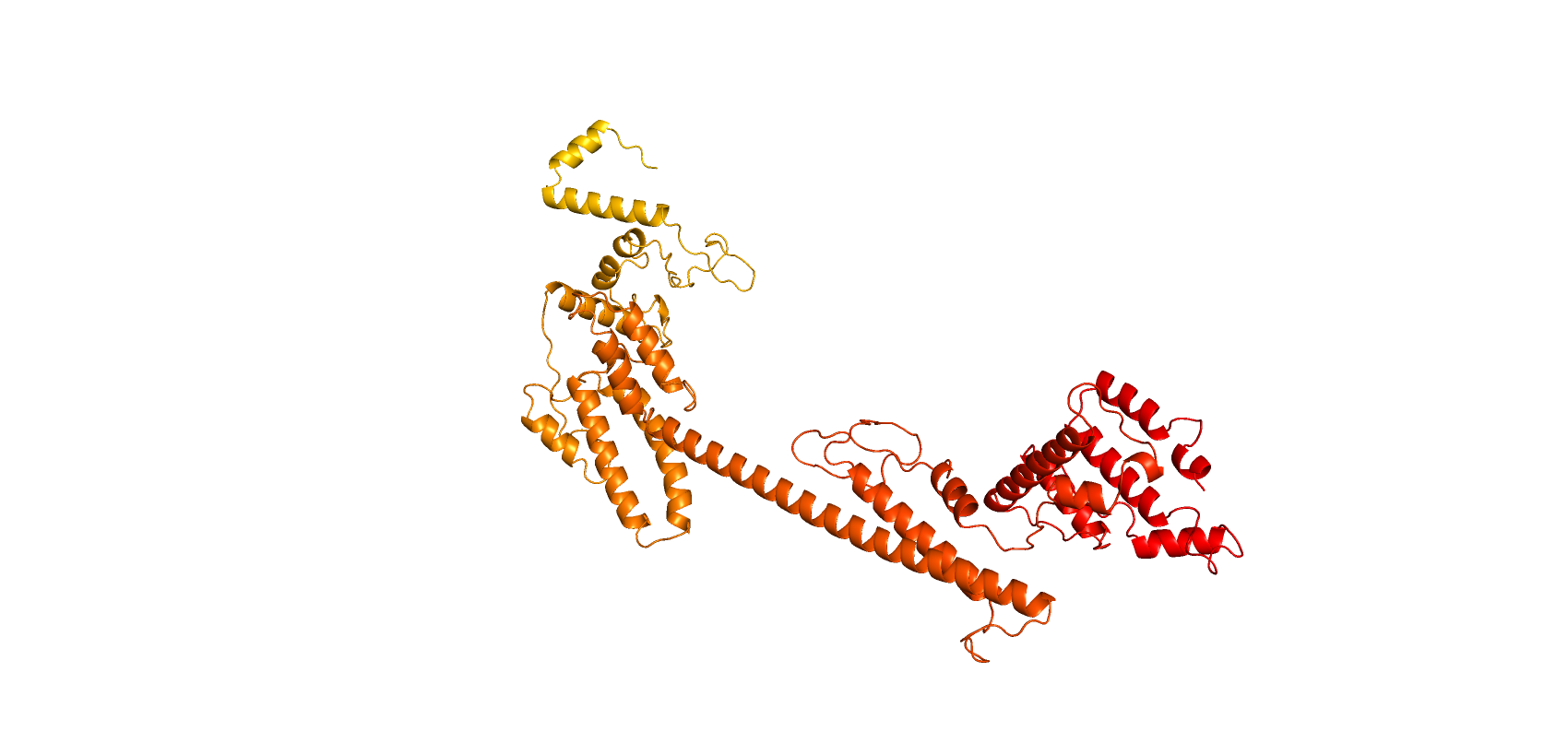 |
| RoseTTAfold | 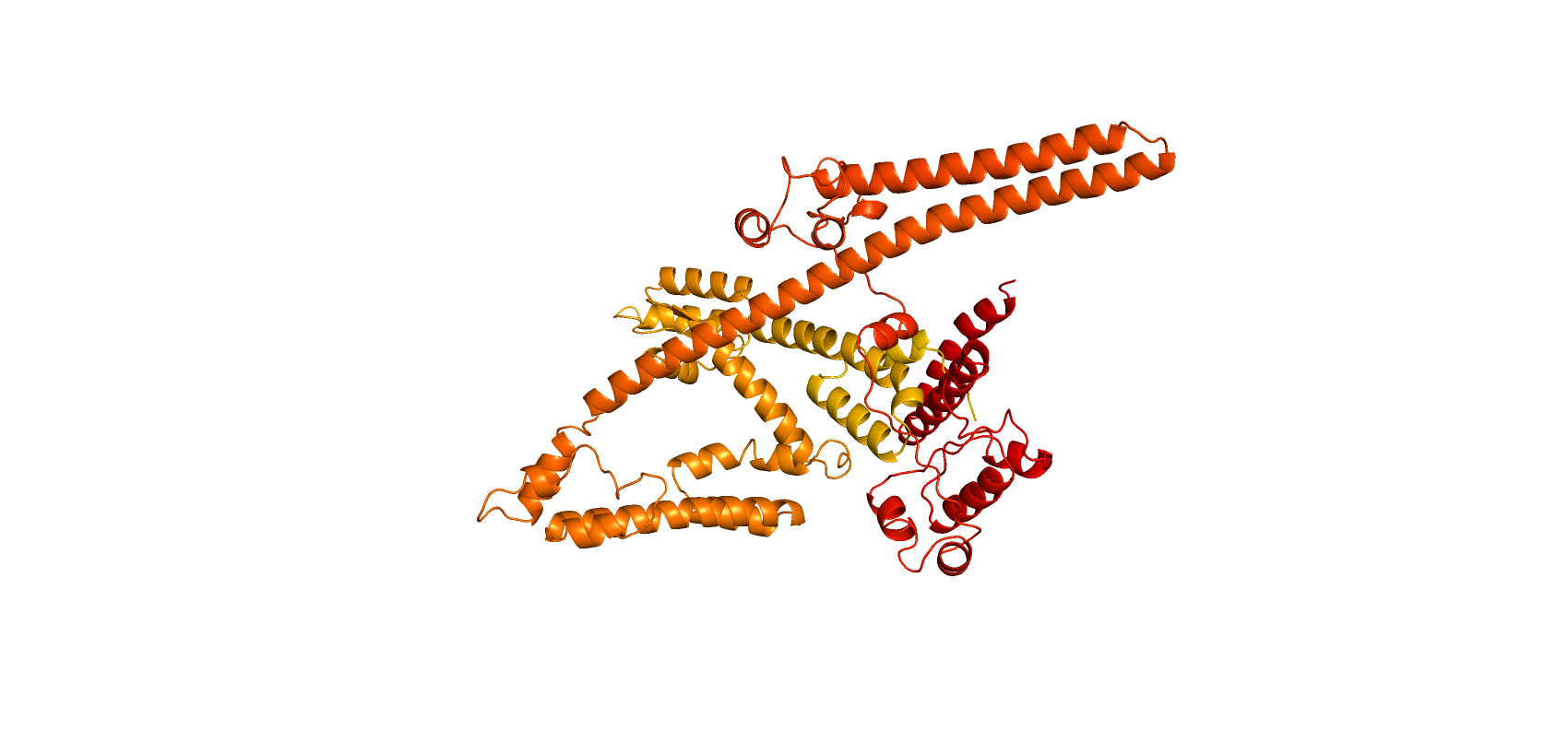 | 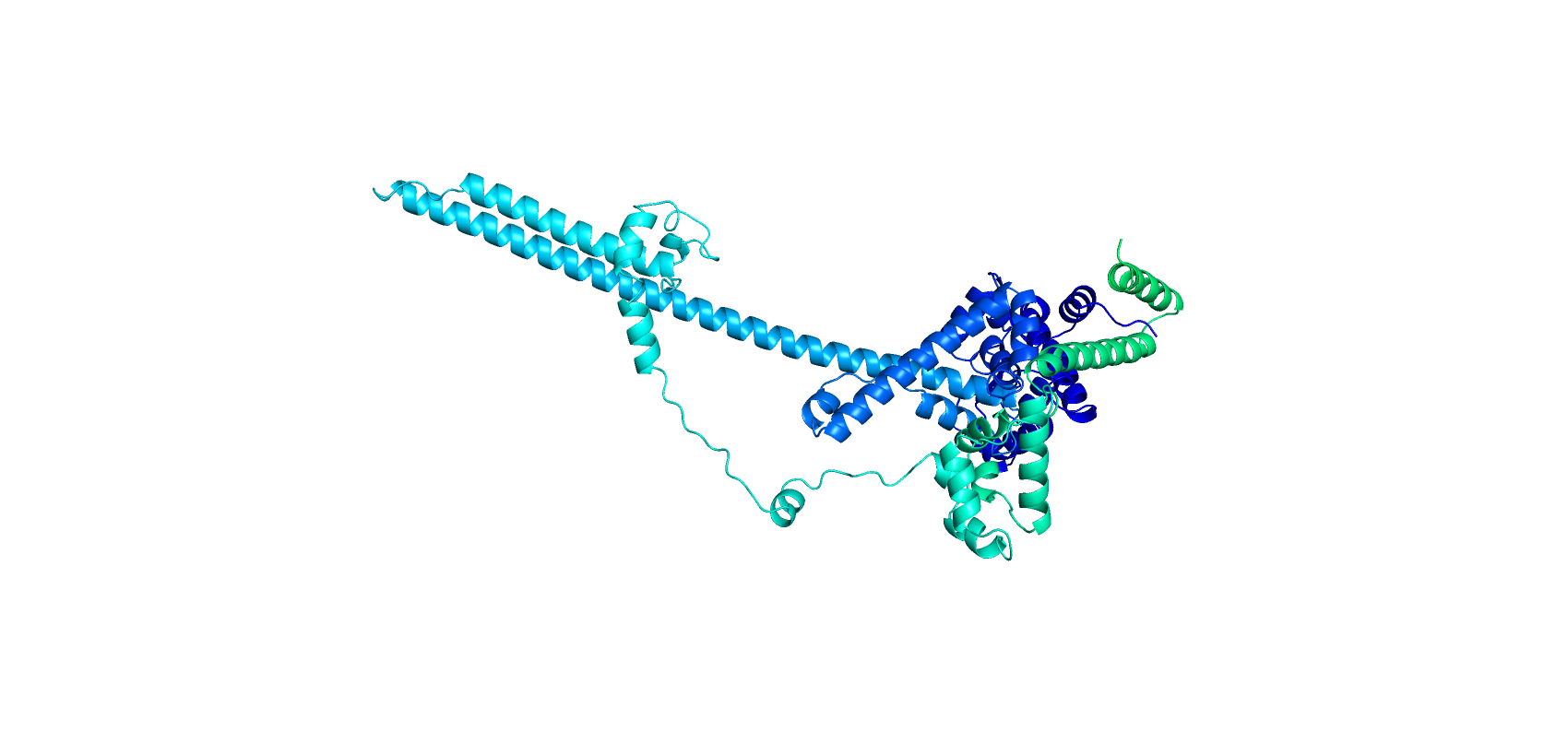 |

**Supplementary information 6.** Complete list of refined tertiary structures of vaccine constructs.

| **Vaxign2 (Galaxy Refine)** | | | | | |
| --- | --- | --- | --- | --- | --- |
| **BA. 1** | | | | | |
| **AlphaFold 2** | | | | | |
| **Model** | **Residues in most favoured regions** | **Residues in additional allowed regions** | **Residues in generously allowed regions** | **Residues in disallowed regions** | **Z-score (ProSA-web)** |
| Model 1 | 482 (94.9%) | 22 (4.3%) | 2 (0.4%) | 2 (0.4%) | -4.97 |
| Model 2 | 486 (95.7%) | 19 (3.7%) | 0 (0%) | 3 (0.6%) | -4.82 |
| Model 3 | 485 (95.5%) | 20 (3.9%) | 2 (0.4%) | 1 (0.2%) | -4.73 |
| Model 4 | 485 (95.5%) | 20 (3.9%) | 0 (0%) | 3 (0.6%) | -4.88 |
| Model 5 | 485 (95.5%) | 20 (3.9%) | 1 (0.2%) | 2 (0.4%) | -4.8 |
| **RaptorX** | | | | | |
| **Model** | **Residues in most favoured regions** | **Residues in additional allowed regions** | **Residues in generously allowed regions** | **Residues in disallowed regions** | **Z-score (ProSA-web)** |
| Model 1 | 454 (89.4%) | 48 (9.4%) | 3 (0.6%) | 3 (0.6%) | -7.38 |
| Model 2 | 457 (90%) | 45 (8.9%) | 3 (0.6%) | 3 (0.6%) | -7.44 |
| Model 3 | 456 (89.8%) | 47 (9.3%) | 2 (0.4%) | 3 (0.6%) | -7.22 |
| Model 4 | 453 (89.2%) | 49 (9.6%) | 2 (0.4%) | 4 (0.8%) | -7.61 |
| Model 5 | 459 (90.4%) | 44 (8.7%) | 1 (0.2%) | 4 (0.8%) | -7.29 |
| **RoseTTAfold** | | | | | |
| **Model** | **Residues in most favoured regions** | **Residues in additional allowed regions** | **Residues in generously allowed regions** | **Residues in disallowed regions** | **Z-score (ProSA-web)** |
| Model 1 | 479 (94.3%) | 22 (4.3%) | 1 (0.2%) | 6 (1.2%) | -7.86 |
| Model 2 | 479 (94.3%) | 21 (4.1%) | 2 (0.4%) | 6 (1.2%) | -8.16 |
| Model 3 | 476 (93.7%) | 24 (4.7%) | 2 (0.4%) | 6 (1.2%) | -8.13 |
| Model 4 | 479 (94.3%) | 21 (4.1%) | 2 (0.4%) | 6 (1.2%) | -7.95 |
| Model 5 | 476 (93.7%) | 25 (4.9%) | 1 (0.2%) | 6 (1.2%) | -8.2 |
| **BA.2** | | | | | |
| **AlphaFold 2** | | | | | |
| **Model** | **Residues in most favoured regions** | **Residues in additional allowed regions** | **Residues in generously allowed regions** | **Residues in disallowed regions** | **Z-score (ProSA-web)** |
| Model 1 | 482 (95.1%) | 23 (4.5%) | 0 (0%) | 2 (0.4%) | -4.7 |
| Model 2 | 484 (95.5%) | 19 (3.7%) | 3 (0.6%) | 1 (0.2%) | -4.65 |
| Model 3 | 483 (95.3%) | 21 (4.1%) | 2 (0.4%) | 1 (0.2%) | -4.45 |
| Model 4 | 478 (94.3%) | 27 (5.3%) | 0 (0%) | 2 (0.4%) | -4.63 |
| Model 5 | 486 (95.9%) | 19 (3.7%) | 9 (0%) | 2 (0.4%) | -4.64 |
| **RaptorX** | | | | | |
| **Model** | **Residues in most favoured regions** | **Residues in additional allowed regions** | **Residues in generously allowed regions** | **Residues in disallowed regions** | **Z-score (ProSA-web)** |
| Model 1 | 458 (90.3%) | 46 (9.1%) | 1 (0.2%) | 2 (0.4%) | -8.32 |
| Model 2 | 463 (91.3%) | 41 (8.1%) | 2 (0.4%) | 1 (0.2%) | -8.32 |
| Model 3 | 459 (90.5%) | 44 (8.7%) | 3 (0.6%) | 1 (0.2%) | -8.24 |
| Model 4 | 457 (90.1%) | 47 (9.3%) | 1 (0.2%) | 2 (0.4%) | -8.25 |
| Model 5 | 460 (90.7%) | 44 (8.7%) | 2 (0.4%) | 1 (0.2%) | -8.3 |
| **RoseTTAfold** | | | | | |
| **Model** | **Residues in most favoured regions** | **Residues in additional allowed regions** | **Residues in generously allowed regions** | **Residues in disallowed regions** | **Z-score (ProSA-web)** |
| Model 1 | 473 (93.3%) | 27 (5.3%) | 2 (0.4%) | 5 (1%) | -8.94 |
| Model 2 | 472 (93.1%) | 27 (5.3%) | 1 (0.2%) | 7 (1.4%) | -8.88 |
| Model 3 | 470 (92.7%) | 29 (5.7%) | 3 (0.6%) | 5 (1%) | -8.86 |
| Model 4 | 471 (92.9%) | 28 (5.5%) | 2 (0.4%) | 6 (1.2%) | -8.76 |
| Model 5 | 470 (92.7%) | 30 (5.9%) | 0 (0%) | 7 (1.4%) | -8.91 |
| **IEDB (Galaxy Refine)** | | | | | |
| **BA. 1** | | | | | |
| **AlphaFold 2** | | | | | |
| **Model** | **Residues in most favoured regions** | **Residues in additional allowed regions** | **Residues in generously allowed regions** | **Residues in disallowed regions** | **Z-score (ProSA-web)** |
| Model 1 | 460 (91.5%) | 33 (6.6%) | 4 (0.8%) | 6 (1.2%) | -3.76 |
| Model 2 | 469 (93.2%) | 27 (5.4%) | 5 (1%) | 2 (0.4%) | -3.87 |
| Model 3 | 465 (92.4%) | 30 (6%) | 5 (1%) | 3 (0.6%) | -3.86 |
| Model 4 | 459 (91.3%) | 36 (7.2%) | 7 (1.4%) | 1 (0.2%) | -3.92 |
| Model 5 | 464 (92.2%) | 30 (6%) | 4 (0.8%) | 5 (1%) | -3.91 |
| **RaptorX** | | | | | |
| **Model** | **Residues in most favoured regions** | **Residues in additional allowed regions** | **Residues in generously allowed regions** | **Residues in disallowed regions** | **Z-score (ProSA-web)** |
| Model 1 | 436 (86.7%) | 59 (11.7%) | 5 (1%) | 3 (0.6%) | -6.41 |
| Model 2 | 440 (87.5%) | 56 (11.1%) | 3 (0.6%) | 4 (0.8%) | -6.33 |
| Model 3 | 436 (86.7%) | 58 (11.5%) | 5 (1%) | 4 (0.8%) | -6.25 |
| Model 4 | 435 (86.5%) | 59 (11.7%) | 6 (1.2%) | 3 (0.6%) | -6.35 |
| Model 5 | 437 (86.9%) | 58 (11.5%) | 5 (1%) | 3 (0.6%) | -6.19 |
| **RoseTTAfold** | | | | | |
| **Model** | **Residues in most favoured regions** | **Residues in additional allowed regions** | **Residues in generously allowed regions** | **Residues in disallowed regions** | **Z-score (ProSA-web)** |
| Model 1 | 470 (93.4%) | 24 (4.8%) | 4 (0.8%) | 5 (1%) | -8.49 |
| Model 2 | 468 (93%) | 26 (5.2%) | 3 (0.6%) | 6 (1.2%) | -8.55 |
| Model 3 | 472 (93.8%) | 23 (4.6%) | 3 (0.6%) | 5 (1%) | -8.59 |
| Model 4 | 467 (92.8%) | 27 (5.4%) | 5 (1%) | 4 (0.8%) | -8.51 |
| Model 5 | 466 (92.6%) | 27 (5.4%) | 6 (1.2%) | 4 (0.8%) | -8.58 |
| **BA.2** | | | | | |
| **AlphaFold 2** | | | | | |
| **Model** | **Residues in most favoured regions** | **Residues in additional allowed regions** | **Residues in generously allowed regions** | **Residues in disallowed regions** | **Z-score (ProSA-web)** |
| Model 1 | 482 (96%) | 17 (3.4%) | 1 (0.2%) | 2 (0.4%) | -5.17 |
| Model 2 | 482 (96%) | 16 (3.2%) | 1 (0.2%) | 3 (0.6%) | -5.33 |
| Model 3 | 481 (95.8%) | 16 (3.2%) | 3 (0.6%) | 2 (0.4%) | -5.46 |
| Model 4 | 477 (95%) | 21 (4.2%) | 0 (0%) | 4 (0.8%) | -5.09 |
| Model 5 | 478 (95.2%) | 19 (3.8%) | 2 (0.4%) | 3 (0.6%) | -5.31 |
| **RaptorX** | | | | | |
| **Model** | **Residues in most favoured regions** | **Residues in additional allowed regions** | **Residues in generously allowed regions** | **Residues in disallowed regions** | **Z-score (ProSA-web)** |
| Model 1 | 455 (90.6%) | 36 (7.2%) | 7 (1.4%) | 4 (0.8%) | -8.15 |
| Model 2 | 452 (90%) | 40 (8%) | 7 (1.4%) | 3 (0.6%) | -8 |
| Model 3 | 455 (90.6%) | 37 (7.4%) | 7 (1.4%) | 3 (0.6%) | -8.14 |
| Model 4 | 456 (90.8%) | 38 (7.6%) | 5 (1%) | 3 (0.6%) | -8.11 |
| Model 5 | 453 (90.2%) | 39 (7.8%) | 6 (1.2%) | 4 (0.8%) | -8.18 |
| **RoseTTAfold** | | | | | |
| **Model** | **Residues in most favoured regions** | **Residues in additional allowed regions** | **Residues in generously allowed regions** | **Residues in disallowed regions** | **Z-score (ProSA-web)** |
| Model 1 | 469 (93.4%) | 20 (4%) | 7 (1.4%) | 6 (1.2%) | -9.21 |
| Model 2 | 467 (93%) | 22 (4.4%) | 7 (1.4%) | 6 (1.2%) | -9.09 |
| Model 3 | 470 (93.6%) | 20 (4%) | 6 (1.2%) | 6 (1.2%) | -9.08 |
| Model 4 | 470 (93.6%) | 20 (4%) | 6 (1.2%) | 6 (1.2%) | -9.42 |
| Model 5 | 468 (93.2%) | 21 (4.2%) | 8 (1.6%) | 5 (1%) | -9.19 |

**Supplementary information 7.** Interface residues between ACE2 receptor and the vaccine constructs (all models).

| **Vaxign 2** | | | | | | | | |
| --- | --- | --- | --- | --- | --- | --- | --- | --- |
| **BA. 1** | | | | | | | | |
| **Model** | **Interface area, Å2** | **ΔiG, kcal/mol** | **ΔiG, P-value** |  | **Hydrogen bonds** | | | |
| AlphaFold 2 | 2762.6 | -22.7 | 0.96 |  | **#** | **Structure 1** | **Dist. [Å]** | **Structure 2** |
|  |  |  |  |  | 1 | A:LYS 353[ HZ1] | 1.77 | B:GLU 426[ OE1] |
|  |  |  |  |  | 2 | A:LYS 353[ HZ2] | 1.73 | B:GLU 426[ OE2] |
|  |  |  |  |  | 3 | A:ASN  53[HD21] | 2.06 | B:PRO 432[ O  ] |
|  |  |  |  |  | 4 | A:GLN 305[HE21] | 1.90 | B:ALA 465[ O  ] |
|  |  |  |  |  | 5 | A:LEU 333[ H  ] | 2.13 | B:LYS 467[ O  ] |
|  |  |  |  |  | 6 | A:GLN 305[HE22] | 2.04 | B:GLY 468[ O  ] |
|  |  |  |  |  | 7 | A:LYS 363[ HZ2] | 1.84 | B:ALA 525[ O  ] |
|  |  |  |  |  | 8 | A:LYS 363[ HZ1] | 1.73 | B:GLU 526[ OE2] |
|  |  |  |  |  | 9 | A:LYS 363[ HZ2] | 2.35 | B:GLU 526[ O  ] |
|  |  |  |  |  | 10 | A:LYS 341[ HZ1] | 1.81 | B:ALA 529[ O  ] |
|  |  |  |  |  | 11 | A:LYS 131[ HZ1] | 1.69 | B:GLN 534[ OE1] |
|  |  |  |  |  | 12 | A:TYR  50[ HH ] | 2.27 | B:TYR 552[ O  ] |
|  |  |  |  |  | 13 | A:TYR 515[ HH ] | 2.36 | B:LEU 564[ O  ] |
|  |  |  |  |  | 14 | A:ARG 514[HH22] | 2.20 | B:PHE 566[ O  ] |
|  |  |  |  |  | 15 | A:TYR 515[ HH ] | 1.88 | B:ILE 567[ O  ] |
|  |  |  |  |  | 16 | A:GLU 329[ OE1] | 1.79 | B:LYS 396[ HZ3] |
|  |  |  |  |  | 17 | A:GLU 329[ OE2] | 1.76 | B:LYS 396[ HZ2] |
|  |  |  |  |  | 18 | A:TYR  41[ OH ] | 2.11 | B:ALA 428[ H  ] |
|  |  |  |  |  | 19 | A:ASN  53[ OD1] | 2.00 | B:ARG 434[ HE ] |
|  |  |  |  |  | 20 | A:VAL 339[ O  ] | 2.23 | B:ARG 434[HH11] |
|  |  |  |  |  | 21 | A:ILE  54[ O  ] | 2.02 | B:ARG 434[HH22] |
|  |  |  |  |  | 22 | A:ASN  53[ OD1] | 1.79 | B:ARG 434[HH21] |
|  |  |  |  |  | 23 | A:ASP 295[ OD1] | 2.26 | B:SER 521[ H  ] |
|  |  |  |  |  | 24 | A:GLU 145[ OE1] | 1.86 | B:LYS 530[ HZ3] |
|  |  |  |  |  | 25 | A:GLU 145[ OE2] | 1.79 | B:LYS 530[ HZ2] |
|  |  |  |  |  | 26 | A:ASP 335[ OD2] | 2.10 | B:ARG 532[ HE ] |
|  |  |  |  |  | 27 | A:GLU  56[ OE2] | 1.94 | B:TYR 552[ HH ] |
|  |  |  |  |  | 28 | A:THR 125[ O  ] | 2.31 | B:LYS 557[ HZ3] |
|  |  |  |  |  | 29 | A:THR 129[ OG1] | 1.80 | B:LYS 557[ HZ3] |
|  |  |  |  |  | 30 | A:SER 124[ O  ] | 1.92 | B:TYR 561[ HH ] |
| RaptorX | 1523.1 | -4.7 | 0.709 |  | **#** | **Structure 1** | **Dist. [Å]** | **Structure 2** |
|  |  |  |  |  | 1 | A:ARG 357[HH11] | 1.29 | B:GLU 405[ OE1] |
|  |  |  |  |  | 2 | A:MET  82[ SD ] | 2.05 | B:ARG 495[HH11] |
| RoseTTAFold | 1174.1 | -11.5 | 0.797 |  | **#** | **Structure 1** | **Dist. [Å]** | **Structure 2** |
|  |  |  |  |  | 1 | A:LYS  31[ HZ3] | 1.72 | B:PHE 438[ O  ] |
|  |  |  |  |  | 2 | A:GLY 326[ H  ] | 2.00 | B:GLN 554[ OE1] |
|  |  |  |  |  | 3 | A:ASN 330[HD22] | 1.94 | B:TYR 555[ OH ] |
|  |  |  |  |  | 4 | A:ASP  30[ OD2] | 1.79 | B:ARG 439[HH12] |
|  |  |  |  |  | 5 | A:GLU  35[ OE1] | 1.75 | B:LYS 467[ HZ2] |
|  |  |  |  |  | 6 | A:GLU 329[ OE1] | 1.96 | B:LYS 551[ HZ3] |
|  |  |  |  |  | 7 | A:ASN 330[ OD1] | 1.67 | B:LYS 551[ HZ1] |
| **BA.2** | | | | | | | | |
| **Model** | **Interface area, Å2** | **ΔiG, kcal/mol** | **ΔiG, P-value** |  | **Hydrogen bonds** | | | |
| AlphaFold 2 | 2690 | -11.5 | 0.934 |  | **#** | **Structure 1** | **Dist. [Å]** | **Structure 2** |
|  |  |  |  |  | 1 | A:ASN  64[HD22] | 2.28 | B:PHE 147[ O  ] |
|  |  |  |  |  | 2 | A:GLN  60[HE21] | 2.06 | B:PHE 147[ O  ] |
|  |  |  |  |  | 3 | A:GLN  60[HE22] | 2.41 | B:GLU 148[ OE1] |
|  |  |  |  |  | 4 | A:ASN  63[HD21] | 2.13 | B:ALA 150[ O  ] |
|  |  |  |  |  | 5 | A:ASN 117[HD21] | 2.41 | B:ALA 151[ O  ] |
|  |  |  |  |  | 6 | A:SER 105[ H  ] | 2.27 | B:ASN 155[ OD1] |
|  |  |  |  |  | 7 | A:ASN 394[HD21] | 1.99 | B:LEU 159[ O  ] |
|  |  |  |  |  | 8 | A:ASN  51[HD22] | 2.03 | B:GLU 163[ OE2] |
|  |  |  |  |  | 9 | A:LYS 353[ HZ3] | 1.77 | B:ASP 375[ OD1] |
|  |  |  |  |  | 10 | A:SER  19[ OG ] | 3.39 | B:TYR 500[ O  ] |
|  |  |  |  |  | 11 | A:GLU 329[ OE1] | 1.78 | B:LYS  74[ HZ1] |
|  |  |  |  |  | 12 | A:GLU 329[ OE2] | 1.83 | B:LYS  74[ HZ2] |
|  |  |  |  |  | 13 | A:THR 334[ OG1] | 1.66 | B:LYS  91[ HZ3] |
|  |  |  |  |  | 14 | A:GLN  60[ OE1] | 1.66 | B:LYS 145[ HZ2] |
|  |  |  |  |  | 15 | A:GLU  56[ OE2] | 1.77 | B:LYS 145[ HZ3] |
|  |  |  |  |  | 16 | A:ASP  67[ OD2] | 2.06 | B:ALA 151[ H  ] |
|  |  |  |  |  | 17 | A:ASP  67[ OD2] | 2.05 | B:LYS 152[ H  ] |
|  |  |  |  |  | 18 | A:GLU 110[ OE1] | 1.91 | B:LYS 152[ HZ1] |
|  |  |  |  |  | 19 | A:GLU 110[ OE2] | 1.65 | B:LYS 152[ HZ3] |
|  |  |  |  |  | 20 | A:GLU 110[ O  ] | 2.12 | B:LYS 152[ HZ2] |
|  |  |  |  |  | 21 | A:ASP  67[ OD1] | 2.04 | B:PHE 153[ H  ] |
|  |  |  |  |  | 22 | A:SER 105[ OG ] | 2.42 | B:ASN 155[ H  ] |
|  |  |  |  |  | 23 | A:SER 124[ OG ] | 1.63 | B:LYS 167[ HZ2] |
|  |  |  |  |  | 24 | A:SER 124[ O  ] | 2.24 | B:LYS 167[ HZ3] |
|  |  |  |  |  | 25 | A:SER 128[ OG ] | 1.69 | B:LYS 167[ HZ3] |
|  |  |  |  |  | 26 | A:GLU 145[ OE1] | 1.76 | B:LYS 168[ HZ1] |
|  |  |  |  |  | 27 | A:GLU 145[ OE2] | 1.76 | B:LYS 168[ HZ3] |
|  |  |  |  |  | 28 | A:GLU  56[ OE1] | 1.86 | B:ARG 177[HH22] |
|  |  |  |  |  | 29 | A:GLU  37[ OE2] | 1.84 | B:ARG 436[HH21] |
|  |  |  |  |  | 30 | A:GLU  37[ O  ] | 2.07 | B:ARG 436[HH22] |
|  |  |  |  |  | 31 | A:LYS  31[ O  ] | 1.71 | B:PHE 440[ H  ] |
|  |  |  |  |  | 32 | A:GLU  75[ OE1] | 1.88 | B:ARG 441[HH22] |
|  |  |  |  |  | 33 | A:ASP  30[ OD1] | 1.80 | B:ARG 497[HH22] |
|  |  |  |  |  | 34 | A:SER  19[ OG ] | 2.12 | B:VAL 502[ H  ] |
| RaptorX | 1212.9 | -3.4 | 0.968 |  | **#** | **Structure 1** | **Dist. [Å]** | **Structure 2** |
|  |  |  |  |  | 1 | A:LYS  31[ HZ3] | 1.72 | B:LEU 435[ O  ] |
|  |  |  |  |  | 2 | A:LYS  31[ HZ1] | 1.74 | B:ARG 436[ O  ] |
|  |  |  |  |  | 3 | A:GLN  76[HE22] | 1.91 | B:TYR 438[ OH ] |
|  |  |  |  |  | 4 | A:SER  19[ H  ] | 2.07 | B:THR 443[ O  ] |
|  |  |  |  |  | 5 | A:GLU  35[ OE1] | 1.89 | B:ARG 436[ HE ] |
|  |  |  |  |  | 6 | A:HIS  34[ O  ] | 2.25 | B:ARG 436[HH22] |
|  |  |  |  |  | 7 | A:GLU  75[ OE1] | 1.92 | B:TYR 438[ HH ] |
|  |  |  |  |  | 8 | A:SER  19[ OG ] | 1.83 | B:ARG 441[HH11] |
|  |  |  |  |  | 9 | A:ASP  30[ OD1] | 2.17 | B:LYS 452[ HZ2] |
|  |  |  |  |  | 10 | A:GLU  37[ OE2] | 2.08 | B:THR 478[ H  ] |
|  |  |  |  |  | 11 | A:GLU  37[ OE2] | 3.71 | B:THR 478[ OG1] |
|  |  |  |  |  | 12 | A:ALA 387[ O  ] | 3.31 | B:SER 481[ OG ] |
|  |  |  |  |  | 13 | A:GLU 329[ OE1] | 2.12 | B:ALA 509[ H  ] |
|  |  |  |  |  | 14 | A:GLN 325[ OE1] | 1.65 | B:LYS 511[ HZ1] |
|  |  |  |  |  | 15 | A:GLU 329[ OE2] | 1.76 | B:LYS 511[ HZ2] |
| RoseTTAFold | 1512.9 | -12.8 | 0.86 |  | **#** | **Structure 1** | **Dist. [Å]** | **Structure 2** |
|  |  |  |  |  | 1 | A:HIS  34[ NE2] | 3.47 | B:LYS 256[ O  ] |
|  |  |  |  |  | 2 | A:TYR  83[ HH ] | 1.85 | B:THR 475[ O  ] |
|  |  |  |  |  | 3 | A:LYS  68[ HZ1] | 1.91 | B:GLU 549[ OE1] |
|  |  |  |  |  | 4 | A:LYS  68[ HZ2] | 1.85 | B:GLU 549[ OE2] |
|  |  |  |  |  | 5 | A:GLN 305[HE21] | 2.12 | B:PHE 568[ O  ] |
|  |  |  |  |  | 6 | A:ALA 301[ O  ] | 2.04 | B:LYS  91[ H  ] |
|  |  |  |  |  | 7 | A:GLN 300[ O  ] | 1.78 | B:LYS  94[ HZ3] |
|  |  |  |  |  | 8 | A:GLU  23[ OE1] | 1.75 | B:LYS 241[ HZ3] |
|  |  |  |  |  | 9 | A:MET  82[ SD ] | 2.30 | B:ASN 479[ H  ] |
|  |  |  |  |  | 10 | A:GLN  42[ OE1] | 1.63 | B:LYS 553[ HZ1] |
| **IEDB** | | | | | | | | |
| **BA. 1** | | | | | | | | |
| **Model** | **Interface area, Å2** | **ΔiG, kcal/mol** | **ΔiG, P-value** |  | **Hydrogen bonds** | | | |
| AlphaFold 2 | 1822.4 | -23.3 | 0.637 |  | **#** | **Structure 1** | **Dist. [Å]** | **Structure 2** |
|  |  |  |  |  | 1 | A:ARG 115[HH21] | 1.88 | B:GLU 521[ OE1] |
|  |  |  |  |  | 2 | A:ARG 115[HH11] | 2.34 | B:GLU 521[ OE1] |
|  |  |  |  |  | 3 | A:ASN 338[HD22] | 1.86 | B:GLN 549[ OE1] |
|  |  |  |  |  | 4 | A:LYS 341[ HZ1] | 1.68 | B:GLN 549[ O  ] |
|  |  |  |  |  | 5 | A:LYS 341[ HZ2] | 1.67 | B:TYR 556[ OH ] |
|  |  |  |  |  | 6 | A:TYR 510[ HH ] | 1.98 | B:PHE 561[ O  ] |
|  |  |  |  |  | 7 | A:ASP 136[ O  ] | 2.06 | B:ASN 140[HD22] |
|  |  |  |  |  | 8 | A:ASP 136[ OD1] | 1.82 | B:LYS 143[ HZ1] |
|  |  |  |  |  | 9 | A:ASP 136[ OD2] | 1.74 | B:LYS 143[ HZ2] |
|  |  |  |  |  | 10 | A:ASN 137[ OD1] | 1.92 | B:ARG 144[HH12] |
|  |  |  |  |  | 11 | A:ASP 136[ OD2] | 2.27 | B:ARG 144[HH22] |
|  |  |  |  |  | 12 | A:ASP 136[ O  ] | 1.84 | B:ARG 144[HH22] |
|  |  |  |  |  | 13 | A:GLU 171[ OE2] | 2.03 | B:ARG 527[ HE ] |
|  |  |  |  |  | 14 | A:SER 170[ O  ] | 1.83 | B:ARG 527[HH22] |
| RaptorX | 1852.1 | -7.4 | 0.956 |  | **#** | **Structure 1** | **Dist. [Å]** | **Structure 2** |
|  |  |  |  |  | 1 | A:ARG 559[HH11] | 1.84 | B:SER 298[ O  ] |
|  |  |  |  |  | 2 | A:ARG 559[HH21] | 1.87 | B:SER 298[ O  ] |
|  |  |  |  |  | 3 | A:ARG 559[HH12] | 2.09 | B:GLU 299[ OE2] |
|  |  |  |  |  | 4 | A:GLN 325[HE21] | 1.93 | B:THR 335[ O  ] |
|  |  |  |  |  | 5 | A:LYS 353[ HZ2] | 1.71 | B:PRO 416[ O  ] |
|  |  |  |  |  | 6 | A:LYS  68[ HZ2] | 1.81 | B:GLU 421[ OE2] |
|  |  |  |  |  | 7 | A:ASN 556[ OD1] | 1.72 | B:ARG 295[HH12] |
|  |  |  |  |  | 8 | A:ASN 556[ OD1] | 1.84 | B:ARG 295[HH22] |
|  |  |  |  |  | 9 | A:MET 383[ O  ] | 1.91 | B:GLN 306[HE22] |
|  |  |  |  |  | 10 | A:ALA 386[ O  ] | 1.94 | B:ARG 309[HH12] |
|  |  |  |  |  | 11 | A:ALA 386[ O  ] | 1.95 | B:ARG 309[HH22] |
|  |  |  |  |  | 12 | A:GLU 329[ OE2] | 2.00 | B:GLN 318[HE22] |
|  |  |  |  |  | 13 | A:GLU 329[ O  ] | 2.12 | B:ARG 328[ H  ] |
|  |  |  |  |  | 14 | A:GLN 305[ OE1] | 1.88 | B:ARG 328[ HE ] |
|  |  |  |  |  | 15 | A:TRP 328[ O  ] | 2.08 | B:ARG 328[HH11] |
|  |  |  |  |  | 16 | A:GLU 329[ OE1] | 2.13 | B:ILE 330[ H  ] |
|  |  |  |  |  | 17 | A:GLU 329[ OE1] | 2.07 | B:ALA 331[ H  ] |
|  |  |  |  |  | 18 | A:GLU 329[ OE1] | 3.86 | B:PRO 332[ N  ] |
|  |  |  |  |  | 19 | A:TYR  83[ OH ] | 1.77 | B:ARG 490[HH12] |
|  |  |  |  |  | 20 | A:GLU 110[ OE2] | 1.74 | B:ARG 506[HH12] |
|  |  |  |  |  | 21 | A:GLU 110[ OE1] | 1.78 | B:ARG 506[HH22] |
|  |  |  |  |  | 22 | A:GLN  24[ OE1] | 1.93 | B:TYR 550[ HH ] |
| RoseTTAFold | 1543.7 | -10.7 | 0.824 |  | **#** | **Structure 1** | **Dist. [Å]** | **Structure 2** |
|  |  |  |  |  | 1 | A:ARG 559[HH21] | 1.76 | B:ASP 388[ OD2] |
|  |  |  |  |  | 2 | A:GLN  42[HE22] | 2.10 | B:GLU 402[ OE1] |
|  |  |  |  |  | 3 | A:LYS 353[ HZ1] | 1.83 | B:GLU 402[ OE2] |
|  |  |  |  |  | 4 | A:LYS 353[ HZ2] | 2.12 | B:GLU 402[ OE2] |
|  |  |  |  |  | 5 | A:GLN  42[ OE1] | 1.76 | B:LYS 406[ HZ3] |
|  |  |  |  |  | 6 | A:ASP  30[ OD2] | 1.82 | B:LYS 425[ HZ3] |
|  |  |  |  |  | 7 | A:TYR  83[ OH ] | 2.30 | B:LEU 428[ H  ] |
|  |  |  |  |  | 8 | A:GLU  75[ OE1] | 1.78 | B:ARG 429[HH12] |
|  |  |  |  |  | 9 | A:GLN  81[ O  ] | 1.89 | B:TYR 431[ HH ] |
|  |  |  |  |  | 10 | A:GLU  87[ OE1] | 1.76 | B:ARG 434[HH22] |
| **BA.2** | | | | | | | | |
| **Model** | **Interface area, Å2** | **ΔiG, kcal/mol** | **ΔiG, P-value** |  | **Hydrogen bonds** | | | |
| AlphaFold 2 | 2053.1 | -19 | 0.916 |  | **#** | **Structure 1** | **Dist. [Å]** | **Structure 2** |
|  |  |  |  |  | 1 | A:GLN  86[HE21] | 2.06 | B:ILE 469[ O  ] |
|  |  |  |  |  | 2 | A:GLN  86[HE22] | 2.26 | B:THR 470[ O  ] |
|  |  |  |  |  | 3 | A:GLN  86[HE22] | 2.27 | B:GLY 472[ O  ] |
|  |  |  |  |  | 4 | A:ARG 559[HH12] | 1.89 | B:ALA 504[ O  ] |
|  |  |  |  |  | 5 | A:ARG 559[HH21] | 1.85 | B:HIS 507[ O  ] |
|  |  |  |  |  | 6 | A:ARG 559[HH11] | 1.87 | B:HIS 507[ O  ] |
|  |  |  |  |  | 7 | A:LYS 353[ HZ3] | 1.75 | B:ASP 515[ OD1] |
|  |  |  |  |  | 8 | A:LYS 353[ HZ2] | 1.81 | B:ASP 515[ OD2] |
|  |  |  |  |  | 9 | A:SER  19[ H  ] | 1.89 | B:TYR 549[ OH ] |
|  |  |  |  |  | 10 | A:GLN  24[HE22] | 1.99 | B:ILE 553[ O  ] |
|  |  |  |  |  | 11 | A:ASP 213[ OD2] | 1.96 | B:ARG 487[ HE ] |
|  |  |  |  |  | 12 | A:ASP 216[ OD2] | 1.86 | B:ARG 487[HH12] |
|  |  |  |  |  | 13 | A:ASP 216[ OD2] | 1.85 | B:ARG 487[HH22] |
|  |  |  |  |  | 14 | A:ASP 213[ OD1] | 2.27 | B:ARG 492[ HE ] |
|  |  |  |  |  | 15 | A:ASP 213[ O  ] | 2.07 | B:TYR 495[ H  ] |
|  |  |  |  |  | 16 | A:GLN 552[ OE1] | 1.87 | B:ARG 508[HH12] |
|  |  |  |  |  | 17 | A:ASN 556[ OD1] | 2.31 | B:ARG 508[HH21] |
|  |  |  |  |  | 18 | A:ASN 556[ OD1] | 2.40 | B:ARG 508[HH22] |
|  |  |  |  |  | 19 | A:GLN 552[ OE1] | 1.84 | B:ARG 508[HH22] |
|  |  |  |  |  | 20 | A:ASP  30[ OD2] | 2.12 | B:ALA 522[ H  ] |
|  |  |  |  |  | 21 | A:GLU  23[ OE2] | 2.22 | B:ARG 529[HH12] |
|  |  |  |  |  | 22 | A:GLU  23[ OE1] | 1.90 | B:ARG 529[HH11] |
|  |  |  |  |  | 23 | A:SER  19[ O  ] | 2.01 | B:TYR 549[ HH ] |
| RaptorX | 944.3 | -4.7 | 0.904 |  | **#** | **Structure 1** | **Dist. [Å]** | **Structure 2** |
|  |  |  |  |  | 1 | A:LYS  68[ HZ1] | 1.66 | B:TYR 439[ OH ] |
|  |  |  |  |  | 2 | A:LYS  31[ HZ3] | 1.71 | B:GLY 440[ O  ] |
|  |  |  |  |  | 3 | A:TYR  41[ HH ] | 1.97 | B:GLN 551[ OE1] |
|  |  |  |  |  | 4 | A:LYS 353[ HZ3] | 1.65 | B:TYR 552[ OH ] |
|  |  |  |  |  | 5 | A:ARG 559[HH21] | 2.35 | B:GLY 562[ O  ] |
|  |  |  |  |  | 6 | A:ARG 559[HH11] | 2.09 | B:GLY 562[ O  ] |
|  |  |  |  |  | 7 | A:ARG 559[HH21] | 1.87 | B:PHE 563[ O  ] |
|  |  |  |  |  | 8 | A:ARG 559[HH21] | 2.21 | B:ILE 564[ O  ] |
|  |  |  |  |  | 9 | A:ARG 559[HH11] | 1.86 | B:ILE 564[ O  ] |
|  |  |  |  |  | 10 | A:ASP  30[ OD1] | 2.04 | B:ARG 431[ HE ] |
|  |  |  |  |  | 11 | A:GLN  96[ OE1] | 1.90 | B:ARG 431[HH12] |
|  |  |  |  |  | 12 | A:ASP  30[ OD2] | 1.78 | B:ARG 431[HH21] |
| RoseTTAFold | 1618 | -13 | 0.846 |  | **#** | **Structure 1** | **Dist. [Å]** | **Structure 2** |
|  |  |  |  |  | 1 | A:ARG 559[HH11] | 1.79 | B:LYS 379[ O  ] |
|  |  |  |  |  | 2 | A:ALA 387[ H  ] | 2.04 | B:GLY 380[ O  ] |
|  |  |  |  |  | 3 | A:GLN 325[ H  ] | 2.01 | B:GLU 384[ OE2] |
|  |  |  |  |  | 4 | A:LYS 353[ HZ3] | 1.72 | B:GLU 394[ OE1] |
|  |  |  |  |  | 5 | A:LYS 353[ HZ2] | 1.78 | B:GLU 394[ OE2] |
|  |  |  |  |  | 6 | A:LYS  31[ HZ3] | 1.69 | B:THR 403[ O  ] |
|  |  |  |  |  | 7 | A:GLN  24[HE22] | 2.08 | B:THR 413[ O  ] |
|  |  |  |  |  | 8 | A:ILE  21[ H  ] | 1.94 | B:GLU 415[ OE1] |
|  |  |  |  |  | 9 | A:ASP 216[ OD2] | 1.73 | B:LYS 211[ HZ2] |
|  |  |  |  |  | 10 | A:ASN 210[ OD1] | 1.69 | B:LYS 211[ HZ3] |
|  |  |  |  |  | 11 | A:ASN 556[ OD1] | 1.84 | B:TYR 288[ HH ] |
|  |  |  |  |  | 12 | A:GLN 325[ OE1] | 1.65 | B:LYS 388[ HZ3] |
